# Pathogenicity & virulence of *Mycoplasma hyopneumoniae*

**DOI:** 10.1080/21505594.2020.1842659

**Published:** 2020-12-08

**Authors:** Fernanda M. A. Leal Zimmer, Jéssica Andrade Paes, Arnaldo Zaha, Henrique Bunselmeyer Ferreira

**Affiliations:** a Laboratório de Genômica Estrutural e Funcional, Centro de Biotecnologia, Universidade Federal do Rio Grande Do Sul (UFRGS), Porto Alegre, Brazil; b Programa de Pós-Graduação em Biologia Celular e Molecular, Centro de Biotecnologia, UFRGS, Porto Alegre, Brazil; c Laboratório de Biologia Molecular de Cestódeos, Centro de Biotecnologia, UFRGS, Porto Alegre, Brazil; d Departamento de Biologia Molecular e Biotecnologia, Instituto de Biociências, UFRGS, Porto Alegre, Brazil

**Keywords:** *Mycoplasma hyopneumoniae*, porcine enzootic pneumonia, pathogenicity, virulence factors, host response

## Abstract

*Mycoplasma hyopneumoniae*: is the etiological agent of porcine enzootic pneumonia (EP), a disease that impacts the swine industry worldwide. Pathogen-induced damage, as well as the elicited host-response, contribute to disease. Here, we provide an overview of EP epidemiology, control and prevention, and a more in-depth review of *M. hyopneumoniae* pathogenicity determinants, highlighting some molecular mechanisms of pathogen-host interactions relevant for pathogenesis. Based on recent functional, immunological, and comparative “omics” results, we discuss the roles of many known or putative *M. hyopneumoniae* virulence factors, along with host molecules involved in EP. Moreover, the known molecular bases of pathogenicity mechanisms, including *M. hyopneumoniae* adhesion to host respiratory epithelium, protein secretion, cell damage, host microbicidal response and its modulation, and maintenance of *M. hyopneumoniae* homeostasis during infection are described. Recent findings regarding *M. hyopneumoniae* pathogenicity determinants also contribute to the development of novel diagnostic tests, vaccines, and treatments for EP.

## Introduction

*Mycoplasma hyopneumoniae* is the etiological agent of porcine enzootic pneumonia (EP), a chronic respiratory disease that affects pigs [1]. Infections with *M. hyopneumoniae* are highly prevalent worldwide, causing major economic losses to the swine industry [2] due to costs of treatment and vaccination, decreased feed conversion rate, and increased mortality resulting from secondary infections [3]. Immunodiagnosis of *M. hyopneumoniae* infections and vaccination against EP are also challenging due to the still limited repertoire of well-characterized antigens. Some diagnostic and/or vaccinal antigens have been characterized immunologically, i.e., with an assessment of their antigenicity and immunogenicity [4–8], and functionally, with an assessment of their roles in *M. hyopneumoniae* physiology and pathogen-host interactions [9–15].

In the context of the damage-response framework of microbial pathogenesis [16], EP pathology is mainly determined by the damage caused by interactions between *M. hyopneumoniae* and the swine host. It is known that *M. hyopneumoniae* infection leads to epithelial damage of the swine respiratory tract, either directly, considering possible bacterial cytotoxicity [14], or indirectly, by causing a strong and damaging inflammatory response. The damage is usually restricted to the bacterium-caused loss of cilia and cell death, and those caused by the host inflammatory response against the pathogen [2]. The exact mechanisms underlying the immunopathogenesis of *M. hyopneumoniae*, however, are not clear. It is reasonable to assume that the onset of EP in swine depends on *M. hyopneumoniae* pathogenicity determinants, classically described as virulence factors, as well as on the triggered host responses, immunological or otherwise. *M. hyopneumoniae* pathogenicity determinants are considered the bacterial factors that allow the pathogen to override the host defense mechanisms and comprehend molecules that mediate processes such as cell adhesion to the host, response to host environment stress, and immunomodulation. On the other hand, host factors contributing to EP include molecules and processes that mediate innate and adaptive defenses against the pathogen, but also cause damage host tissues, such as lung lesions resulting from exacerbated inflammatory responses.

In the last decade, comparative analyses between pathogenic and nonpathogenic *M. hyopneumoniae* strains, and between *M. hyopneumoniae* and the closely related species *Mycoplasma flocculare*, have considerably improved the knowledge of *M. hyopneumoniae* pathogenesis. *M. hyopneumoniae* strains may differ in pathogenicity or virulence, ranging from the nonpathogenic type strain J (ATCC 25,934) to virulent strains isolated from EP outbreaks, such as the Brazilian strain 7448 or the American strain 232 [17–20]. *M. flocculare*, in turn, is genetically closely related to *M. hyopneumoniae* but is a commensal species, with its presence in the host being virtually asymptomatic. The “omics” prospective and comparative studies with these strains and species, discussed in a later section, have improved our knowledge on the factors that determine *M. hyopneumoniae* pathogenicity.

Host EP determinants, on the other hand, have been less assessed, aside from some punctual studies regarding innate and adaptive immunological response and cell death [21–24]. Nevertheless, differences between pathogenic and nonpathogenic strains or closely related species may also depend on the triggered host responses. Recently, comparative studies regarding the interactions between *M. hyopneumoniae* or *M. flocculare* and the swine host demonstrated differences in intracellular and secreted protein repertoires of swine cells infected by these mycoplasma species [25,26].

This review includes recent data regarding EP epidemiology, prevention, and control of this disease, and a comprehensive overview of the main *M. hyopneumoniae* and swine molecular mechanisms and cellular processes underlying the disease. Moreover, it also surveys recent advances toward the identification of both the bacterial and host repertoires of EP determinants, including the most recent efforts involving functional and comparative “omics” approaches to elucidate why some *M. hyopneumoniae* strains are more virulent than others and why *M. flocculare* is nonpathogenic despite of sharing most of the virulence factors with *M. hyopneumoniae*. Interactions between *M. hyopneumoniae* and swine cells are discussed in the context of their intimate contact during infection, emphasizing molecular/cellular mechanisms related to cell adhesion, biofilm formation, host cell invasion, secretion and signaling, cytotoxicity and apoptosis, immunomodulation and stress response.

## Epidemiology, prevention and control of EP

### Epidemiology

As far as is known, *M. hyopneumoniae* is a specific pathogen of domestic pigs (*Sus scrofa domesticus*) and wild boars (*Sus scrofa scrofa*), and has a worldwide geographical distribution [27]. Specific data on *M. hyopneumoniae* prevalence by country are scarce in the literature, as EP does not require mandatory notification in many countries and does not limit commercial trade [2]. An average *M. hyopneumoniae* prevalence of 30–80% has been reported in domestic pig herds worldwide [28]. In South America, the estimated *M. hyopneumoniae* prevalence was 48% for pigs in the Mendoza province, Argentina, based on molecular diagnosis [29], and prevalence varying from 52% (based on serology of non-vaccinated animals) to more than 90% (based on molecular diagnosis of slaughtered animals) was reported for Southeastern and Southern Brazil [30,31]. A lower *M. hyopneumoniae* prevalence, near 10%, was reported for pigs in Africa (Uganda) [32]. For wild boars, the most recent studies carried out in European countries, such as Sweden and Italy, have shown seroprevalences of *M. hyopneumoniae* of 24.8% and 21.12%, respectively [33,34]. In the last decade, the wild boar population has increased in Europe, which increases the likelihood of its potential contact with domestic pigs, and, thus, the risk of transmission of *M. hyopneumoniae* and other pathogens [34].

*Mycoplasma hyopneumoniae* transmission dynamics within swine herds depend on the intensity of the production system used, as recently reviewed by Maes et al. [2]. The first exposure events occur during the lactation period, when piglets are in contact with dams shedding the microorganism [35,36]. At weaning age, colonization with *M. hyopneumoniae* is important in segregated production systems, since pigs are transferred to clean facilities for the growing and finishing phases [37,38]. Subsequent transmission of *M. hyopneumoniae* among pen-mates is slow, and a clear understanding of *M. hyopneumoniae* transmission in the field is still needed to improve infection models used in experimental researches [39–41]. A critical aspect of the epidemiology of *M. hyopneumoniae* is its long pathogen persistence [42,43], but the factors that determine such persistence are still poorly understood.

An additional complicating factor regarding *M. hyopneumoniae* epidemiology is the occurrence of distinct strains, with different degrees of virulence, circulating in the field [2]. Therefore, the identification and characterization of the *M. hyopneumoniae* strains circulating within a herd or geographical region are of utmost importance. Discrimination among *M. hyopneumoniae* strains usually rely on partial sequencing of the P146 gene [45], multilocus sequence typing (MLST) [46], and multiple-locus variable number tandem repeat analysis (MLVA) [47–49]. More recently, Betlach et al. [49] reviewed published information on *M. hyopneumoniae* variability in pathogenicity and at the antigenic, proteomic, transcriptomic, and genomic levels, and proposed the variable number of tandem repeats (VNTR)-based common terminology and classification. This VNTR-based system is expected to avoid discrepancies and allow to make inferences across the literature.

### Prevention and control

Enzootic pneumonia control and prevention are based on the optimization of management conditions, vaccination and treatment with antibiotics. Factors such as management, biosecurity practices and housing conditions should be optimized within the herd [2]. Decrease of infection levels and/or improvement of the clinical outcome of *M. hyopneumoniae* infections can be achieved with strategies involving management practices such as all-in/all-out pig flows, medicated and segregated early weaning, and multisite operations [27]. Preventing strategies to avoid the introduction of the pathogen in farms free of *M. hyopneumoniae* are also important. Garza-Moreno *et al.* [50] presented different *M. hyopneumoniae* monitoring strategies of incoming gilts and recipient herds and proposed a farm classification based on their health status. According to clinical signs, lung lesions, and ELISA and PCR results, farms and incoming replacements can then be classified into negative, provisional negative and positive.

Vaccination is today still regarded as the most effective way to control *M. hyopneumoniae* infections. Gilt replacement acclimation procedures against *M. hyopneumoniae* in positive farms in Europe and North America showed that vaccination is the main strategy to avoid EP [50]. Anti-*M. hyopneumoniae* vaccines are used worldwide and consist mainly of inactivated, adjuvanted whole-cell preparations that are administered intramuscularly [27]. Presently, there are at least 26 vaccines approved and commercially available worldwide to prevent *M. hyopneumoniae* infection [51].

Vaccinated animals present reduced clinical signs and lung lesions, improved performance and reduced number of microorganisms in the respiratory tract [2,38,52,53]. Although vaccination confers overall beneficial effects in most infected herds, the results are often variable [54–56]. These variations in the outcomes of vaccination may be due to many factors, including different infection levels, diversity of the circulating *M. hyopneumoniae* strains, and unknown aspects of the induced immune responses, along with technical issues, such as improper vaccine storage conditions and administration, and lack of vaccination compliance [44,57]. Furthermore, thus far, commercially available anti-*M. hyopneumoniae* vaccines have conferred only a limited reduction in the transmission ratios [41,58].

The exact mechanisms of protection needed to avoid *M. hyopneumoniae* infection are not yet fully understood, but constant efforts are invested in the development of new vaccines that may confer better protection. The latest efforts toward the development of more efficient vaccines against EP have been recently reviewed by Tao *et al.* [51]. These efforts focus on genetically engineered vaccines and some novel combined vaccines. The most recent genetically engineered vaccines are based on adhesins, such as P97, P95, P46, P42, and P36 delivered as recombinant vectors or recombinant subunits [8,59–62]. However, of a total of 24 genetically engineered vaccines studied over the years, only eleven were tested for their efficacies in pigs.

As *M. hyopneumoniae* infection can predispose swine to secondary infections, combined vaccines have gained attention, as they prevent multiple diseases at the same time. Combined vaccines developed thus far for EP and other swine respiratory infections consist of a mixture of *M. hyopneumoniae* bacterin and live attenuated viruses, such as porcine reproductive and respiratory syndrome virus and porcine circovirus 2 (PCV2) [63–65], or genetically engineered antigens from *M. hyopneumoniae* and other porcine pathogens [66]. There have been studies demonstrating the efficacy of a combined vaccine against *M. hyopneumoniae* and PCV2 [61,67], and their promising results suggest that bivalent or multivalent vaccines may present advantages over monovalent vaccines.

When EP control by the improvement of management and biosecurity and the implementation of vaccination fails, the clinical disease occurs. Then, the treatment of affected animals with antibiotics is required to maintain animal health and welfare. Treatment of *M. hyopneumoniae* infections can be accomplished using medication with antibiotics against *M. hyopneumoniae* and major secondary invading bacteria [68]. Potentially active antibiotics against *M. hyopneumoniae* include tetracyclines, macrolides, lincosamides, pleuromutilins, amphenicols, aminoglycosides, aminocyclitols and fluoroquinolones [27,69]. *M. hyopneumoniae* strains with high minimal inhibitory concentration (MIC) values for some antibiotics have been reported, along with a description of resistance mechanisms [70,71]. Therefore, even with the use of antibiotics, improvements in management and/or housing conditions are still necessary to ensure long-lasting effects during and after antimicrobial treatment.

## *M. hyopneumoniae* “omics” studies

The “omics” era started for *M. hyopneumoniae* in 2004, when the first strains (the pathogenic strains 232 and 7448, and the nonpathogenic type strain J) had their whole genomes sequenced [18,19]. Since then, the genomes of several other strains have been sequenced, and, presently, 21 whole sequenced *M. hyopneumoniae* genomes are available, 10 already assembled and annotated, and 11 still not fully assembled (https://www.ncbi.nlm.nih.gov/genome/browse#!/prokaryotes/190/). Apart that of *M. hyopneumoniae* J strain, the available sequenced genomes are from American, Brazilian, European, Korean, and Chinese pathogenic strains. These genomes are 0.86–0.96 Mb in size, and in each of them there are 528 to 691 protein-encoding genes. Interestingly, despite their small sizes, up to 30% of their gene contents are still of unknown function [72]. Moreover, 20 to 30% of the *M. hyopneumoniae* genes code for surface proteins (many of them also of unknown function) [72], therefore pointing out to a complex and still poorly characterized scenario of pathogen-host interactions.

More recently, functional “omics” studies, including transcriptomic, proteomic, and metabolomic, have followed the pioneering descriptive and comparative genomics research. These studies were enriched by data generated for the close relative species *M. flocculare* and have provided insights into *M. hyopneumoniae* pathogenicity determinants. *M. hyopneumoniae* strains and *M. flocculare* share most (>85%) of the genes that code for known (or predicted as such) pathogenicity determinants, including most adhesins, proteases and antioxidant proteins [19,72]. Moreover, they also share at least 90% of the genes coding for surface proteins. These findings have raised questions regarding the gene products and mechanisms that effectively underlie the differences in pathogenicity or virulence between *M. hyopneumoniae* strains, and between them and *M. flocculare*. Therefore, comparative studies between *M. hyopneumoniae* and *M. flocculare* have focused on possible differences in gene expression and protein abundances. Transcriptomics, proteomics, secretomics, and metabolomics approaches have been carried out as attempts to correlate differential *M. hyopneumoniae* transcripts, proteins or metabolites to pathogenicity or virulence [73–76]. In the following sections the main findings of these functional, comparative “omics” studies are integrated with those from several other complementary studies and discussed, to provide an overview of *M. hyopneumoniae* virulence factors and both bacterial and host EP determinants.

## *M. hyopneumoniae* adhesion to swine epithelial cells

**Figure 1.**
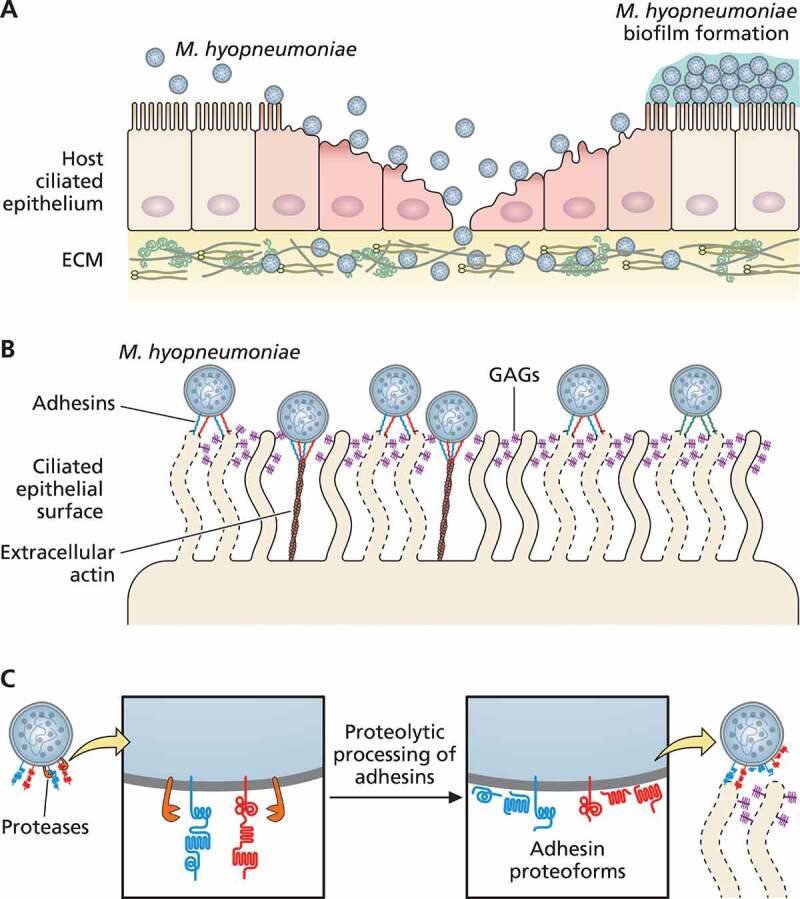
**Schematic representation of *M. hyopneumoniae* adhesion to swine ciliated respiratory epithelium**. (a) *M. hyopneumoniae* cells attach to the cilia of respiratory epithelium, causing ciliostasis, cilium loss, and subsequent epithelial cell death. *M. hyopneumoniae* cells may form biofilms on the ciliated epithelial surface. *M. hyopneumoniae* also interacts with molecules from the ECM, such as fibronectin and plasminogen. (b) *M. hyopneumoniae* adhesion to ciliated cells is mediated by bacterial adhesins that interact with host ligands, as GAGs (displayed on cilia surface), and extracellular actin. (c) Adhesins of *M. hyopneumoniae* are endoproteolytically processed by surface-displayed proteases, generating a combinatorial library of adhesin proteoforms exposed on the bacterial surface. Dashed lines represent damaged ciliated epithelial cells

In EP, *M. hyopneumoniae* adhesion to ciliated epithelial cells of the respiratory tract is the initial event in host colonization. This adhesion process is complex, dynamic, and not fully understood, but it is known to involve several surface-displayed molecules of both the pathogen and the host cells [77,78]. The available information on *M. hyopneumoniae* and host cell adhesion determinants are discussed in the next sub-sections and are schematically represented in Figure 1.

### M. hyopneumoniae *adhesion determinants*

The interaction between pathogenic bacteria and host cells during colonization is a critical process for pathogen survival and disease development [79]. Upon inhalation, *M. hyopneumoniae* must cope with the mucociliary apparatus in the swine respiratory tract, as the so-called lung mucociliary clearance (MCC) is the primary defense mechanism against respiratory pathogens [80]. It is the result of the coordinated interaction of the mucus and a low viscosity periciliary layer with ciliated epithelial cells. The mucus entraps inhaled pathogens and the low viscosity periciliary layer lubricates the airway surface, facilitating the ciliary beating that propels pathogens and particles out of host airways.

*Mycoplasma hyopneumoniae* attachment to the cilia of the respiratory epithelium allows the pathogen to overcome MCC. It has been long known that the pathogen adhesion causes ciliostasis and subsequent cilium loss and epithelial cell death [81,82]. More recently, *in vitro* infection assays provided evidence that *M. hyopneumoniae* infection disrupts the mucociliary function of respiratory epithelial cells by transiently reducing the amounts of mucin 5B secreted in the respiratory epithelium [83]. This reduction causes an uneven distribution of this mucin and might lead to the damage to the epithelial structure, including loss of cilia, observed upon *M. hyopneumoniae* adhesion to ciliated respiratory cells.

A repertoire of at least 35 *M. hyopneumoniae* proteins have been previously associated with cell adhesion, including several related to the P97/P102 paralog families and other surface proteins that moonlight as adhesins [78,84,85] (Table 1). However, the number of *M. hyopneumoniae* adhesins can be higher, considering that its surfaceome includes more than 290 proteins [72,75,86] and that many uncharacterized surface-displayed proteins may bear adhesion properties. Moreover, *M. hyopneumoniae* pathogenic and nonpathogenic strains, and the nonpathogenic *M. flocculare*, which differ in pathogenicity and were classically described as having differential adhesion capacities [87], share almost their entire repertoires of known adhesins, according to more recent comparative genomics and proteomics studies [72,75,87], also pointing out to differences beyond the mere sets of known adhesins.

**Table 1.** *M. hyopneumoniae* surface adhesins and adhesion-related proteins and their host ligands

Protein name	*M. hyopneumoniae* strains ^a^			Host ligands ^b^	Endoproteolytic processing ^c^	References
	232	7448	J			
46 kDa surface antigen (p46)	mhp511	MHP7448_0513	MHJ_0511	Fibronectin, heparin	Y	[78,94]
ABC transporter xylose-binding lipoprotein	mhp623	MHP7448_0604	MHJ_0606	Fibronectin, heparin	Y	[78,94]
Acetate kinase	mhp505	MHP7448_0508	MHJ_0505	Heparin	Y	[78]
Adenine phosphoribosyltransferase	mhp266	MHP7448_0114	MHJ_0110	Fibronectin, heparin	Y	[78]
Adhesin like-protein P146	mhp684	MHP7448_0663	MHJ_0663	Fibronectin, plasminogen, heparin, porcine epithelial cilia	Y	[78,170]
ATP-dependent zinc metalloprotease FtsH	mhp175	MHP7448_0206	MHJ_0202	Fibronectin, heparin	Y	[78]
Chaperone protein DnaK (HSP70)	mhp072	MHP7448_0067	MHJ_0063	Fibronectin, heparin	Y	[78,94]
Dihydrolipoamide dehydrogenase	mhp504	MHP7448_0507	MHJ_0504	Fibronectin, heparin	Y	[78]
Elongation factor Tu (EfTu)	mhp540	MHP7448_0523	MHJ_0524	Fibronectin, heparin	Y	[78,171]
Glyceraldehyde 3-phosphate dehydrogenase	mhp036	MHP7448_0035	MHJ_0031	Fibronectin	Y	[78]
Hexulose-6-phosphate synthase	mhp441	MHP7448_0438	MHJ_0436	Heparin	Y	[78]
L-lactate dehydrogenase (LDH)	mhp245	MHP7448_0137	MHJ_0133	Fibronectin, heparin	Y	[78]
Leucyl aminopeptidase	mhp462	MHP7448_0464	MHJ_0461	Plasminogen, heparin	Y	[10]
Lipoprotein	mhp390	MHP7448_0378	MHJ_0374	Porcine epithelial cilia	NR	[139]
Lppt protein	mhp384	MHP7448_0372	MHJ_0368	Heparin, porcine epithelial cilia	Y	[78,90]
M42 glutamyl aminopeptidase	mhp252	MHP7448_0129	MHJ_0125	Plasminogen, heparin	Y	[95]
Oligoendopeptidase F	mhp520	MHP7448_0521	MHJ_0522	Heparin	Y	[78]
P97-copy 1	mhp183	MHP7448_0198	MHJ_0194	Fibronectin, plasminogen, heparin, porcine epithelial cilia	Y	[78,91,93,94]
P102-copy 1	mhp182	MHP7448_0199	MHJ_0195	Fibronectin, plasminogen, porcine epithelial cilia	Y	[100]
P97-copy 2	mhp271	MHP7448_0108	MHJ_0105	Fibronectin, heparin, porcine epithelial cilia	Y	[98]
P102-copy 2 ^d^	mhp272	MHP7448_0107	MHJ_0104	ND	NR	[18,19]
P97-like protein	mhp107	MHP7448_0272	MHJ_0264	Fibronectin, plasminogen, heparin, porcine epithelial cilia	Y	[99]
P102-like protein	mhp108	MHP7448_0271	MHJ_0263	Fibronectin, plasminogen, porcine epithelial cilia	Y	[110]
Periplasmic sugar-binding protein	mhp145	MHP7448_0234	MHJ_0227	Fibronectin, heparin	Y	[78]
Putative MgpA like-protein ^d^	mhp005	MHP7448_0005	MHJ_0005	ND	NR	[19,172]
Putative P76 membrane protein (P159)	mhp494	MHP7448_0497	MHJ_0494	Fibronectin, heparin, porcine epithelial cilia	Y	[78,173]
Putative P216 surface protein	mhp493	MHP7448_0496	MHJ_0493	Heparin, porcine epithelial cilia	Y	[15,94,102]
Putative prolipoprotein P65	mhp677	MHP7448_0656	MHJ_0656	Fibronectin, heparin	Y	[78]
Pyruvate dehydrogenase	mhp264	MHP7448_0116	MHJ_0112	Fibronectin, heparin	Y	[78]
Pyruvate dehydrogenase E1-alpha subunit	mhp265	MHP7448_0115	MHJ_0111	Fibronectin, heparin	Y	[78]
Uncharacterized protein	mhp009	MHP7448_0009	MHJ_0009	Heparin	Y	[78]
Uncharacterized protein	mhp165	MHP7448_0216	MHJ_0212	Heparin	Y	[78]
Uncharacterized protein	mhp347	MHP7448_0335	MHJ_0326	Fibronectin, heparin	Y	[78]
Uncharacterized protein	mhp385	MHP7448_0373	MHJ_0369	Heparin, porcine epithelial cilia	Y	[78,90]
Uncharacterized protein	mhp683	MHP7448_0662	MHJ_0662	Heparin, porcine epithelial cilia	Y	[78,89]

^a^NCBI accession numbers corresponding to the genes annotated in the *M. hyopneumoniae* 232, 7448 and J (RefSeq NC_006360.1, NC_007332.1 and NC_007295.1, respectively).

^b^Host ligands according to published data available for at least one *M. hyopneumoniae* strain; ND, not determined.

^c^Endoproteolytic processing with published experimental evidence available for at least one *M. hyopneumoniae* strain. Y, yes; NR, not reported.

^d^P102 copy-2 and putative MgpA-like protein adhesins were predicted as such based on their paralogy/orthology with *M. hyopneumoniae* P102 copy-1 and *Mycoplasma genitalium* MgPa adhesins, respectively.

One of the possible explanations for the differences in adhesion capacity between *M. hyopneumoniae* strains and their nonpathogenic counterpart *M. flocculare* would be differences in the amount of adhesins presented at the cell surface. Different adhesins may vary in abundance at the cell surface between mycoplasma strains or species, due to differential transcriptional rates of the respective genes, and/or to differential translational rates of the corresponding mRNAs. However, few differences have been found in the expression of ortholog adhesins from *M. hyopneumoniae* and *M. flocculare* at transcriptional and proteomic levels [75,76]. Taken together, the available comparative transcriptomic and proteomic results suggest that there are no major overall differences in the repertoires of known adhesins between *M. hyopneumoniae* pathogenic and nonpathogenic strains and *M. flocculare* due to gene expression regulation at the transcriptional and translational levels.

Alternative explanations for the observed differences in the adhesion capacity of different *M. hyopneumoniae* strains and *M. flocculare* can be found in post-translational events, including their export to the cell membrane and proteolytic processing. Regarding protein export, the most typical *M. hyopneumoniae* and *M. flocculare* adhesins, such as members of the P97 and P102 adhesin families, have predicted signal-peptides and are likely exported by the general secretory Sec pathway [74]. Other less typical adhesins, such as some enzymes that moonlight as adhesion proteins at the cell surface, lack a signal-peptide and would be exported by non-classic pathways. However, differential export efficiency apparently is not a major determinant of the differences in the abundance of adhesins in the cell surface, as both *M. hyopneumoniae* and *M. flocculare* surfaceomes are similarly enriched with adhesins [75].

Regarding proteolytic processing, it has been described that many adhesins may be targets of endoproteolytic post-translational processing [15,78,88–93] (Table 1). These proteolytic processing events can shape the bacterial surface architecture [78,94], generating several adhesin proteoforms, that may be aimed to different locations and exert alternative functions. At least some of them are displayed at the cell surface, while others may stay in the cytoplasm or be released from the cell membrane to the extracellular milieu. Apart from those adhesin proteoforms with *bona fide* transmembrane domains, it is not known how processed adhesin proteoforms are anchored in the mycoplasma cell membrane. While some of these proteoforms may interact with and be retained by glycosaminoglycans (GAGs) [15,89,90], others may stay only transiently in the cell surface and then be released as soluble secretion products [74].

A recent comparative analysis of endoproteolytic processing between *M. hyopneumoniae* and *M. flocculare* adhesins [94] demonstrated that the five most abundant of them in the *M. hyopneumoniae* pathogenic strain 7448 are differentially processed compared to their corresponding orthologs in the nonpathogenic *M. hyopneumoniae* J strain and *M. flocculare*. Most of the analyzed surface-displayed adhesins from the pathogenic strain were more proteolytically processed (i.e., cleaved at more sites) than the orthologs from the nonpathogenic counterparts, which is consistent with the observed enrichment of several aminopeptidases and endoproteases at the surface of this pathogenic *M. hyopneumoniae* strain [75]. There is also evidence that adhesins can be differentially proteolytically processed in the cytoplasm, during or after their translation, and/or in the cell membrane, during or after their translocation to the cell surface [94]. Moreover, some adhesin proteoforms generated by proteolytic cleavage were observed in both cytoplasmic and surface compartments, indicating that at least some of the involved proteolytic events occur primarily in the cytoplasm, prior to the translocation of the resulting proteoforms to the cell surface.

The differential adhesin proteoforms generated by proteolytic processing may play differential roles. Those retaining adhesive domains, may retain adhesion properties (i.e., their capacity to bind to host cell or extracellular matrix ligand molecules) and contribute to the overall mycoplasma adhesion capacity. Alternatively, proteoforms devoid of adhesive domains may exert moonlight functions. As many of the generated proteoforms are potentially antigenic [94], and may be presented in the cell surface or even secreted as soluble antigens [74], it is likely that they contribute, at least to some degree, to the mycoplasma strategies of immunomodulation or immunoevasion (discussed in Host immune response and immunomodulation during *M. hyopneumoniae* infection). Therefore, the mechanism of post-translational proteolytic processing of adhesins (including proteases and their target adhesins) would be a main *M. hyopneumoniae* pathogenicity determinant, of upmost relevance for EP determination. It would lead to differential presentations of adhesin proteoforms at the cell surface of *M. hyopneumoniae* strains (and *M. flocculare*), resulting in, and explaining, at least in part, the observed differential adhesion capacities of these strains and species.

Besides the canonical adhesins and derived proteoforms, other proteins, with different primary function, may moonlight as adhesins. For instance, some typical cytosolic proteins that are also consistently displayed at the cell surface likely exert alternative functions in this ectopic compartment. They may exert moonlight functions by interacting with host components and contributing to host colonization. Among these surface-displayed cytosolic proteins, there are glycolytic enzymes, proteases, chaperones and translation factors that have been characterized as adhesins in different mycoplasma species, including *M. hyopneumoniae* [10,84,85,95,96]. Interestingly, several of these known moonlighting proteins are overrepresented at the surface of the pathogenic *M. hyopneumoniae* strain 7448 in comparison to the nonpathogenic J strain and *M. flocculare* [75]. This overrepresentation also likely contributes to the differences in adhesion capacity observed among these strains and species.

### Host cell adhesion determinants

The interaction between *M. hyopneumoniae* adhesins (or adhesin proteoforms) and host cells also depends on the corresponding host ligands on the cell surface or in the extracellular matrix (ECM). Preliminary studies demonstrated that some swine ciliary glycolipids could act as receptors for *M. hyopneumoniae* attachment [97], but the bacterial adhesins involved in these pathogen-host interactions remain unknown. In the last decade, it has been demonstrated that adhesive proteoforms from the *M. hyopneumoniae* P97/P102 adhesin family can bind to GAGs from proteoglycans exposed on the cilia surface, which thereby could act as receptors [89,90]. Indeed, adhesins and adhesin proteoforms of the P97/P102 family display short linear motifs enriched in positively charged amino acids, which promote their binding to anionic molecules, such as GAGs and heparin [78,90,93,98–100]

Apart from the cilia-exposed glycans, some swine ECM molecules, such as fibronectin and plasminogen, also provide binding sites for surface adhesins of *M. hyopneumoniae*, contributing to host colonization [78,93,98,101,102]. Moreover, interactions between ECM molecules and bacterial surface proteins have been described for several mycoplasma species, including *Mycoplasma pneumoniae, Mycoplasma gallisepticum* and *Mycoplasma bovis*, among others [103–107]. Interestingly, the fibronectin-binding ability of *M. hyopneumoniae* may mediate the adherence to swine respiratory cilia and can provide a mechanism for host cytoskeleton rearrangements, which may facilitate bacterial internalization [108]. Additionally, the plasminogen-binding ability of *M. hyopneumoniae* may facilitate its traffic via the circulatory system and penetration into host organs, such as liver, kidneys and spleen, from which it has already been isolated [109,110].

More recently, it was also demonstrated that extracellular actin is used as a surface receptor by different proteoforms of *M. hyopneumoniae* P97 adhesin and another 143 proteins, including lipoproteins, glycolytic enzymes, chaperones and translation factors, among others [77]. Interestingly, anti-actin antibodies inhibit 90% of the ability of *M. hyopneumoniae* to adhere and colonize PK-15 swine cells, indicating that extracellular actin is an important receptor for *M. hyopneumoniae* infection. Surface proteins of *M. hyopneumoniae* also interact with other cytoskeletal proteins besides extracellular actin, such as vimentin, keratin, tubulin, myosin, and tropomyosin [77,111].

### *M. hyopneumoniae* biofilm formation, host cell invasion and systemic trafficking across the porcine respiratory epithelium barrier

Direct contact of *M. hyopneumoniae* cells with each other and with host cells or ECM molecules may render the pathogen capable of forming biofilms. Biofilm formation is a strategy used by many bacteria, including other mycoplasma species [112–114], to cope with host immune response or with antimicrobial effects, thereby rendering bacteria extremely adaptive and thus contributing to virulence. Recent studies have shown that at least some *M. hyopneumoniae* strains are indeed capable of forming biofilms, in experimental *in vitro* conditions, on abiotic surfaces or on host cell monolayers, and within the respiratory tract of experimentally infected swine [115,116]. Biofilm formation makes *M. hyopneumoniae* more resistant to antibiotics, at least *in vitro* [116], providing evidence of the importance of biofilm formation for pathogen survival. The molecular interactions and cellular processes underlying *M. hyopneumoniae* biofilm formation are thus far mostly unknown. However, at least *in vitro*, biofilm formation involves the generation of a subpopulation of unstable large cell variants that may contribute to the release of extracellular DNA, essential for forming biofilms on abiotic surfaces [115].

Along with biofilm formation capacity, another valuable strategy for mycoplasma survival and pathogenesis is the ability to invade host cells, which is well described for *Mycoplasma penetrans* and *M. pneumoniae*, for example [117,118]. *M. hyopneumoniae* is usually regarded as an extracellular mycoplasma. However, the demonstration that it binds to fibronectin and plasmin at the site of infection [100,110,119,120], an interaction classically associated to host cell invasion by bacteria [121,122], suggested that it could also penetrate porcine cells. Indeed, it was recently demonstrated that *M. hyopneumoniae* could invade *in vitro*-infected host-derived epithelial cells [108]. Host cell invasion is mediated by endocytic pathways, which are initiated by interactions between mycoplasma surface proteins and host fibronectin and integrin β1. Remarkably, within porcine cells, at least some bacterial cells can survive phagolysosomal fusion and escape into the cytosol, providing evidence of an alternative intracellular form for *M. hyopneumoniae*. Moreover, infected host porcine cells could function as a source of pathogen cells for re-infection, as internalized, dormant *M. hyopneumoniae* could eventually leave to the extracellular environment. In line with that, evidence that *M. hyopneumoniae* may influence the endosomal trafficking and modulate the maturation of early endosomes was provided by proteomic analyses of swine epithelial cells infected with this pathogen [25]. In such a scenario, infected host cells could also function as a reservoir for the traffic of *M. hyopneumoniae* within the respiratory tract, thereby contributing to the chronic infection status.

As it is mostly recovered from trachea and lung lesions from infected pigs, *M. hyopneumoniae* has been considered an exclusive respiratory pathogen [109]. However, at least in experimentally infected pigs, *M. hyopneumoniae* cells have been re-isolated from inner organs, such as liver, spleen, brain, kidneys, and lymph nodes, although at frequencies lower that of the respiratory tract [109,123,124]. These findings demonstrate that *M. hyopneumoniae* is able to disseminate to extrapulmonary sites within the swine host, but the mechanisms that allow the systemic trafficking of the bacterium across the respiratory epithelial barrier remain elusive. One possible mechanism mediating this *M. hyopneumoniae* trafficking might involve its cell invasion capacity, discussed above. As it has been recently demonstrated that *M. hyopneumoniae* could also, at least *in vitro*, invade porcine macrophages and avoid phagocytosis [125], it can be speculated that, by invading these and possibly other immune cells, the bacterium could be spread to other organs.

Evidence of another possible mechanism that could mediate *M. hyopneumoniae* trafficking to alternative host sites was revelead more recently by experiments using an *in vitro* air-liquid culture system of porcine bronchial epithelial cells [83]. With that, it was shown that *M. hyopneumoniae* cells could migrate across the epithelial barrier by the paracellular route, but not by the transcellular route. In this *in vitro* model, *M. hyopneumoniae* reversibly disrupts the tight junctions between epithelial cells, increasing the permeability and damaging the integrity of the epithelial barrier. In line with that, several studies have demonstrated that *M. hyopneumoniae* infection facilitates the activation of plasminogen to plasmin, which contributes to the degradation of several ECM and cellular junction components [10,95,100,110,120]. Such disruption of the porcine respiratory epithelial barrier would contribute to the extrapulmonary dissemination of *M. hyopneumoniae* and to the persistence of infection. Overall, further studies are needed to elucidate how and to what extant *M. hyopneumoniae* disseminates to different extrapulmonary niches in natural infections.

## Protein secretion of *M. hyopneumoniae* and its impact on pathogen-host interactions

Protein secretion is a vital process for all organisms and has a particular role in the pathogenesis of bacterial infections. Gram-negative and Gram-positive bacteria have at least 6 reported secretion/translocation systems [126]. The general secretory (Sec) pathway comprises an essential, ubiquitous and universal export machinery for most proteins that translocate through (soluble secretome) or integrate into (insoluble secretome) the cell membrane [127]. In mycoplasmas, however, the Sec secretory pathway is apparently incomplete, as some genes coding for components of this pathway were not found in their genomes. In the *M. hyopneumoniae* genome, only the genes coding for SecY, SecG, SecD/F, YidC and SecA proteins were identified, and the absence of the SecE protein may indicate an incomplete SecYEG transmembrane channel [18,19,72]. However, the secretome analysis of *M. hyopneumoniae* provided evidences that the Sec secretory pathway is functional, as several detected proteins in the soluble secretome fraction were predicted to be secreted by this secretion pathway [74]. This evidence suggests that other more divergent and still unknown proteins may be part of the Sec pathway, which is consistent with the fact that ~35% of the *M. hyopneumoniae* genome has thus far no functional annotation.

In contrast, most of the detected proteins in the *M. hyopneumoniae* soluble secretome were predicted as secreted by Sec-independent secretion pathways [74]. This finding suggests that *M. hyopneumoniae* may present alternative secretion mechanisms not yet identified due to their lack of conservation with other well-characterized secretion pathways. In line with that, protein secretion in extracellular vesicles was observed in several mycoplasma species [128,129], suggesting that *M. hyopneumoniae* may alternatively use a vesicle system to secrete proteins.

The soluble secretome of *M. hyopneumoniae* can be considered a reservoir of virulence factors (Table 2), being composed of several adhesins, lipoproteins, and nucleases [26,74]. The detection of adhesins in the soluble secretome fraction may result from the extensive proteolytic processing of these proteins on the *M. hyopneumoniae* cell surface (discussed in M. hyopneumoniae *adhesion determinants*), eventually resulting in the release of some adhesin proteoforms in the extracellular milieu. These adhesin proteoforms may act as extracellular antigens that could trigger the host immunological defenses (discussed in Host immune response and immunomodulation during *M. hyopneumoniae* infection). The potential relevance of the *M. hyopneumoniae* secretome for pathogenesis is evident by comparative analyses with *M. flocculare*, the secretome of which is less complex and presents few orthologs of known *M. hyopneumoniae* virulence factors.

**Table 2.** Putative virulence factors found in the *M. hyopneumoniae* secretome and/or surfaceome

Protein name	*M. hyopneumoniae* strain ^a^			Function associated to pathogenicity ^b^	Subcellular localization ^c^	References
	232	7448	J			
46 kDa surface antigen (p46)	mhp511	MHP7448_0513	MHJ_0511	Adhesion	Se/Su	[26,78]
Adhesin like-protein P146	mhp684	MHP7448_0663	MHJ_0663	Adhesion	Se/Su	[26,170]
Aminopeptidase	mhp252	MHP7448_0129	MHJ_0125	Proteolytic processing, immunomodulation, adhesion	Su	[75,95,151]
ATP-dependent protease binding protein	mhp278	MHP7448_0101	MHJ_0098	Heat shock protein, proteolytic processing	Su	[75,78]
ATP-dependent zinc metalloprotease FtsH	mhp175	MHP7448_0206	MHJ_0202	Proteolytic processing, adhesion	Su	[75,78]
Chaperone protein DnaJ	mhp073	MHP7448_0068	MHJ_0064	Chaperone, post-translational processing	Su	[75,169]
Elongation factor Tu (EfTu)	mhp540	MHP7448_0523	MHJ_0524	Immunomodulation, adhesion	Su	[75,156]
Hemolysin C	mhp663	MHP7448_0643	MHJ_0643	Cytotoxicity	Su	[75,174]
Leucyl aminopeptidase	mhp462	MHP7448_0464	MHJ_0461	Proteolytic processing, adhesion	Su	[10,75]
Lipoprotein	mhp164	MHP7448_0217	MHJ_0213	Cytotoxicity	Se/Su	[21,26,75,138]
Lipoprotein	mhp502	MHP7448_0505	MHJ_0502	Cytotoxicity	Se/Su	[21,26,75,138]
Lipoprotein	mhp378	MHP7448_0367	MHJ_0363	Cytotoxicity	Se/Su	[21,74,75,138]
Lipoprotein	mhp345	MHP7448_0333	MHJ_0324	Cytotoxicity	Su	[21,75,138]
Lipoprotein	mhp377	MHP7448_0366	MHJ_0362	Cytotoxicity	Su	[21,75,138]
Lipoprotein	mhp379	MHP7448_0368	MHJ_0364	Cytotoxicity	Su	[21,75,138]
L-lactate dehydrogenase (LDH)	mhp245	MHP7448_0137	MHJ_0133	Adhesion	Se/Su	[26,78]
Lon protease (ATP-dependent protease La)	mhp541	MHP7448_0524	MHJ_0525	Proteolytic processing	Su	[75]
Lppt protein	mhp384	MHP7448_0372	MHJ_0368	Adhesion	Se/Su	[26,74,90]
Membrane nuclease, lipoprotein	mhp597	MHP7448_0580	MHJ_0581	Surface nuclease, cytotoxicity, imunomodulation	Se/Su	[11,26,74]
Oligoendopeptidase F	mhp520	MHP7448_0521	MHJ_0522	Proteolytic processing, immunomodulation, adhesion	Su	[9,75,151]
Outer membrane protein-P95	mhp280	MHP7448_0099	MHJ_0096	Surface antigen	Se/Su	[169; 26, 75]
P102-copy 1	mhp182	MHP7448_0199	MHJ_0195	Adhesion	Se/Su	[26,74,100]
P102-copy 2 ^d^	mhp272	MHP7448_0107	MHJ_0104	Adhesion	Se/Su	[26,74]
p37-like ABC transporter substrate-binding lipoprotein	mhp371	MHP7448_0360	MHJ_0356	Cytotoxicity	Su	[21,75,138]
P60-like lipoprotein	mhp364	MHP7448_0353	MHJ_0348	Cytotoxicity	Se/Su	[21,74,138]
P97-copy 1	mhp183	MHP7448_0198	MHJ_0194	Adhesion	Se/Su	[26,74,93]
P97-copy 2	mhp271	MHP7448_0108	MHJ_0105	Adhesion	Se/Su	[26,74,98]
Phosphopentomutase	mhp221	MHP7448_0161	MHJ_0157	DNA damage response	Su	[175]
Protein GrpE (HSP-70 cofactor)	mhp011	MHP7448_0011	MHJ_0011	Chaperone, post-translational processing	Se/Su	[169; 26, 75]
Putative lipoprotein	mhp390	MHP7448_0378	MHJ_0374	Cytotoxicity	Se/Su	[21,26,138]
Putative lipoprotein	mhp640	MHP7448_0621	MHJ_0622	Cytotoxicity	Su	[21,75,138]
Putative MgpA like-protein ^d^	mhp005	MHP7448_0005	MHJ_0005	Adhesion	Se/Su	[74]
Putative P216 surface protein	mhp493	MHP7448_0496	MHJ_0493	Adhesion	Se/Su	[15,26,102]
Putative P76 membrane protein (P159)	mhp494	MHP7448_0497	MHJ_0494	Adhesion	Se/Su	[26,173]
Putative prolipoprotein P65	mhp677	MHP7448_0656	MHJ_0656	Adhesion	Se/Su	[26,78]
Signal-peptidase I	mhp028	MHP7448_0026	MHJ_0022	Proteolytic processing, citotoxicity	Su	[13,14]
Thiol peroxidase	mhp283	MHP7448_0096	MHJ_0093	Antioxidant protection	Se	[12,26]
Thioredoxin	mhp396	MHP7448_0384	MHJ_0380	Antioxidant protection	Se	[26]
Trigger factor	mhp233	MHP7448_0149	MHJ_0145	Chaperone, post-translational processing	Se/Su	[169; 26, 75]
XAA-PRO aminopeptidase	mhp680	MHP7448_0659	MHJ_0659	Proteolytic processing, immunomodulation	Se	[9,75]

^a^NCBI accession numbers corresponding to the genes annotated in the *M. hyopneumoniae* 232, 7448 and J sequenced genomes (RefSeq NC_006360.1, NC_007332.1 and NC_007295.1, respectively).

^b^Function(s) associated to pathogenicity predicted *in silico* or according to functional data available for at least one *M. hyopneumoniae* strain.

^c^Subcellular localization according to published proteomic data available for at least one *M. hyopneumoniae* strain. Se, secreted; Su, surface-displayed.

In addition to the differences in the virulence factor content, the overall protein content and abundance in *M. hyopneumoniae* and *M. flocculare* secretomes are differential. Both species possess the genes coding for the Sec secretory pathway components, although the partial Sec secretory machinery may be supplemented by unknown proteins not shared by these mycoplasma species, leading to differential secretion efficiencies. Moreover, *M. hyopneumoniae* and *M. flocculare* may use differential secretion signals and secretion pathways, which resulted in the observed differences in secretome contents. More studies are needed to elucidate the secretion mechanisms used by these and other mycoplasma species.

The insoluble secretome (or surfaceome) of *M. hyopneumoniae* also acts as a reservoir of virulence factors (Table 2), which strongly contribute to EP establishment. Proteomic studies focused on surface proteins have demonstrated that this cell fraction is enriched with adhesion proteins (discussed in section 4.1), lipoproteins, and proteases, among others [75,86]. The roles of these proteins in immunomodulation and cell damage are discussed in the following sections.

## Host immune response and immunomodulation during *M. hyopneumoniae* infection

**Figure 2.**
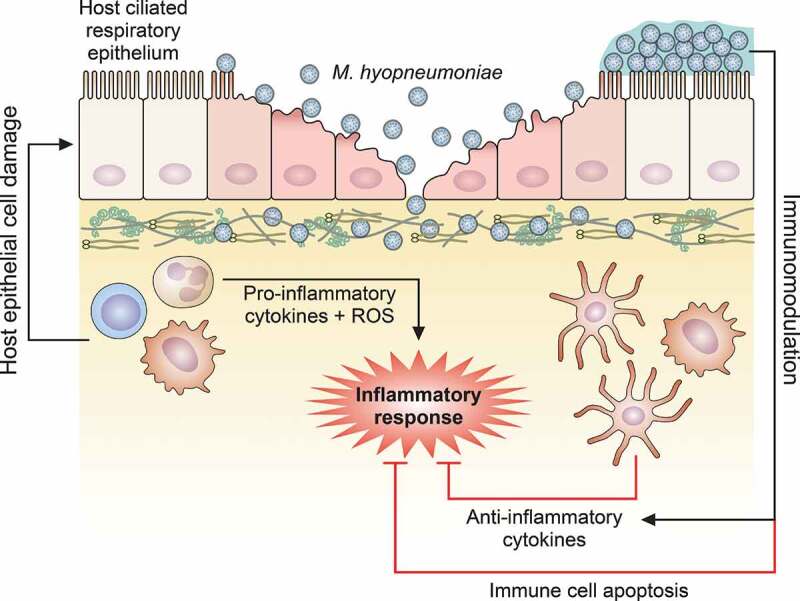
**Schematic representation of host immune response modulation mediated by *M. hyopneumoniae***. *M. hyopneumoniae* infection elicits an acute inflammatory response in swine lungs, represented by a prominent infiltration and accumulation of immune cells that secrete pro-inflammatory cytokines and release ROS, triggering a microbicidal response. However, this pro-inflammatory microbicidal response causes damage to the host respiratory epithelium. Moreover, *M. hyopneumoniae* cells can persist in the respiratory tract modulating the host immune response, eliciting the secretion of anti-inflammatory cytokines by dendritic cells and macrophages, and inducing apoptosis on immune cells accumulated in the respiratory tract. The representation of *M. hyopneumoniae* cells attached to the ciliated respiratory epithelium is described in Figure 1

Upon *M. hyopneumoniae* adherence in the swine respiratory tract (discussed in section 4.1), the host immune response starts to adjust in order to cope with infection. Histopathological changes during *M. hyopneumoniae* infection are frequently observed in the host respiratory tract [130]. They are characterized by a prominent accumulation of mononuclear cells and infiltration of lymphocytes, plasma cells, and neutrophils in the alveolar lumina and septa. Infected lungs present bronchoalveolar exudate, enlargement of alveolar septa and lymphoreticular hyperplasia of the bronchus-associated lymphoid tissue (BALT). Moreover, several studies have demonstrated that the accumulation of immune cells increases the production and secretion of different pro-inflammatory cytokines, including interleukin (IL)-1β, tumor necrosis factor (TNF)-α, IL-6, IL-8, and IL-18 [120,131,132]. The resultant peribronchiolar and perivascular infiltration of mononuclear leukocytes, along with the increase of cytokine production, are, therefore, associated with immunopathological EP lesions. Altogether, these observations support the idea that the host immune cell response against *M. hyopneumoniae* can be considered one of the main causes of the lung lesions observed in EP (Figure 2).

Despite of the acute host inflammatory immune response elicited by *M. hyopneumoniae* infection, EP is considered a chronic disease. The persistence of this pathogen in infected pigs is associated with its capacity to modulate and/or evade of host defenses (Figure 2). This is supported by comparative studies on the porcine immune responses elicited by *M. hyopneumoniae, M. flocculare*, and *M. hyorhinis* (which also colonizes the porcine respiratory tract) revealing that *M. flocculare* and *M. hyorhinis* induce the secretion of higher levels of TNF-α and IL-12, respectively, than *M. hyopneumoniae* [131]. Moreover, *M. hyopneumoniae* infection also induces the expression of high levels of IL-10 but decreases the levels of IL-12 and interferon-γ (IFN-γ) expression in dendritic cells [133]. In line with that, it was demonstrated that a surface protein from *M. hyopneumoniae* elicited the secretion of high levels of IL-10 in murine splenocytes [134]. These findings suggest that *M. hyopneumoniae* infection modulates the host defenses to favor the Th2 immune response, which was also reported in dendritic cells against mycoplasma infection [133], promoting the development of a chronic disease. On the other hand, one study reported a specific systemic humoral immune response found to be predominately involving the IgG2 subclass, suggesting a dominant Th1-mediated immune response to *M. hyopneumoniae* [135]. In fact, there are several inconsistencies regarding the type of predominant immune response induced by *M. hyopneumoniae* [133,135–137]. Such inconsistencies observed in the published literature suggest that *M. hyopneumoniae* can induce mixed Th1/Th2 responses, but more studies are necessary to confirm this dual response and to understand how it is elicited.

The functional modulation of porcine antigen-presenting cells, such as dendritic cells, was also reported in *M. hyopneumoniae* infections [133]. It was demonstrated that *M. hyopneumoniae* down-regulates the expression of the CD1a protein in porcine dendritic cells. Since this protein is responsible for displaying antigenic lipids to T cell receptors, its down-regulation by the pathogen (by a still unknown mechanism), decreases the host dendritic cell capacity for antigen presentation. Moreover, *M. hyopneumoniae* also reduces the overall populations of dendritic and T cells in the porcine nasal cavity in long-term infections, weakening the immune function in the upper respiratory tract [133].

The ability of *M. hyopneumoniae* to reduce the populations of immune cells may be associated with the cytotoxic potential of some bacterial proteins displayed at its surface (Figure 2). *M. hyopneumoniae* cytotoxicity has been considered a virulence mechanism, as the bacterial infection induces apoptosis of immune cells, weakening the immune system, and apoptosis of respiratory epithelial cells, resulting in physical damage to the host tissue. Lipid-associated membrane proteins (LAMPs) from *M. hyopneumoniae* are known to be crucial for *M. hyopneumoniae* cytotoxicity to host cells, as they induce cell death by apoptosis or necrosis and modulate the inflammatory response [21,22,138]. It was demonstrated that these LAMPs induce apoptosis in porcine peripheral blood mononuclear cells, alveolar macrophages and lung epithelial cells *in vitro* by increasing the levels of nitric oxide (NO) and reactive oxygen species (ROS) and by caspase-3 activation. Besides LAMPs, the *M. hyopneumoniae* P68 surface lipoprotein was also identified as an inflammatory and pro-apoptotic mediator to swine immune cells [139]. In this context, apoptosis induction in host immune cells may elicit an immunosuppressive effect, which likely plays an important role in immunomodulation and evasion.

Nitric oxide and ROS are important players in host immune response, contributing to inflammation and microbicidal response. NO is used as a signaling molecule involved in the regulation of vascular hemodynamics and mediates interaction and recruitment of immune cells during infection [140–142]. ROS are key toxic metabolites to kill bacterial pathogens [143]. These molecules are produced intracellularly by phagocytic cells, such as neutrophils and macrophages, eliminating phagocytized bacteria in a process called respiratory burst response [144]. However, bacterial pathogens can subvert these microbicidal responses and induce apoptosis in host cells by increasing the host production and release of NO and ROS [142,145]. Indeed, NO and ROS are also crucial signal molecules for host cell apoptosis, being implicated in the apoptotic cascade and in the activation of initiator and effector caspases [146]. Porcine cells treated with *M. hyopneumoniae* LAMPs presented high levels of NO and superoxide anion radicals, which form peroxynitrite, and lead to oxidative stress and activation of the apoptotic cascade [21,22,138]. This cytopathogenic mechanism was also observed in other mycoplasma species, including *M. hyorhinis, Mycoplasma synoviae*, and *M. pneumoniae* [147–149].

The success of host colonization by several mycoplasma species depends on their ability to rapidly alter the antigenic repertoire of their surface, using different genetic systems, such as those of phase- or antigenic variation caused by DNA slippage [150]. However, for *M. hyopneumoniae*, no evidence of such a mechanism has been found. Instead, the differential proteolytic processing resulting in the presence of differential proteoforms on the *M. hyopneumoniae* surface (discussed in M. hyopneumoniae *adhesion determinants*) may contribute to antigenic variation, which can be associated with immune response modulation and/or evasion. In this sense, *M. hyopneumoniae* surface-associated proteases would be indirectly involved in the modulation of the host immune response, by generating proteoforms of adhesins (and possibly of other surface proteins) presenting different epitope sets.

Furthermore, *M. hyopneumoniae* surface proteases have also been associated with immunomodulation through the proteolytic degradation of pro-inflammatory peptides, such as bradykinin, kininogen, substance P, neurokinin A, and neuropeptide Y [9,151]. It is known that bradykinin and kininogen are involved in the host innate immune response, being associated with bronchoconstriction, MCC and cough induction [152]. Moreover, substance P, neurokinin A and neuropeptide Y are known as inducers of the inflammatory response, eliciting the secretion of high levels of pro-inflammatory cytokines and chemokines [153,154]. Therefore, the proteolytic degradation of these peptides can be considered a virulence mechanism associated with ciliostasis, impairing MCC, and modulation of host immune response.

*Mycoplasma hyopneumoniae* also displays the ability to evade cellular immune responses. Recently, it was demonstrated that *M. hyopneumoniae* evades the phagocytic uptake by porcine alveolar macrophages *in vitro*, although the mechanism underlying this resistance to phagocytosis is still unclear [125]. The presence of convalescent sera, as a source of specific antibodies and complement components for opsonization, did not improve *M. hyopneumoniae* phagocytosis by cell. This result indicates that, although *M. hyopneumoniae* can induce the production of antibodies in the host [155], they are not protective. Another mechanism of host immune evasion by *M. hyopneumoniae* may be the inhibition of the complement pathway. Indeed, it has recently shown that several *M. hyopneumoniae* surface-displayed proteins bind complement factor H, namely elongation factor thermo unstable (EF-Tu), P146, pyruvate dehydrogenase (acetyl-transferring) E1 component subunit alpha (PdhA), P46, pyruvate dehydrogenase E1 component subunit beta (PdhB), glyceraldehyde-3-phosphate dehydrogenase (GAPDH), and three different hypothetical proteins [156]. Factor H binding to the *M. hyopneumoniae* cell surface can confer to the bacterium the ability to inactivate the C3b eventually deposited on the *M. hyopneumoniae* surface, thereby avoiding the consequences of complement activation [157]. The binding of factor H by EF-Tu also contributed to decrease C3 deposition on the *M. hyopneumoniae* surface and increased *M. hyopneumoniae* adhesion to epithelial cells [156]. By counteracting complement activation effects on *M. hyopneumoniae*, these mechanisms contribute to immune evasion and allow more efficient colonization of the respiratory epithelium by the pathogen.

Moreover, it has been shown that *M. hyopneumoniae* is also able to evade neutrophil/macrophage extracellular traps (NETs or METs, respectively), at least *in vitro* [11,158]. In line with that, it has also been demonstrated that the surface nuclease MnuA is responsible for the degradation of NETs, allowing *M. hyopneumoniae* to escape the host immune defense [11].

Overall, despite fostering a strong immune response, *M. hyopneumoniae* can persist for several months in the swine host [42,43]. In this context, the ability of *M. hyopneumoniae* to modulate the host immune responses is an important feature that impacts virulence and disease progression. The entire set of immunomodulation mechanisms used by *M. hyopneumoniae*, however, remains elusive and is worthy of future investigation.

## Cell damage induced by *M. hyopneumoniae* infection

The mechanisms underlying *M. hyopneumoniae* pathogenesis described thus far are bacterium-induced loss of cilia and cell death, which can also be correlated to the damage caused by the intense host microbicidal response against this pathogen [2]. On the other hand, there were additional studies that described the presentation of cytotoxic proteins at the bacterial cell surface and the release of toxic bacterial metabolites as a *M. hyopneumoniae* virulence mechanism causing damage in swine tissues as will be discussed below.

**Figure 3.**
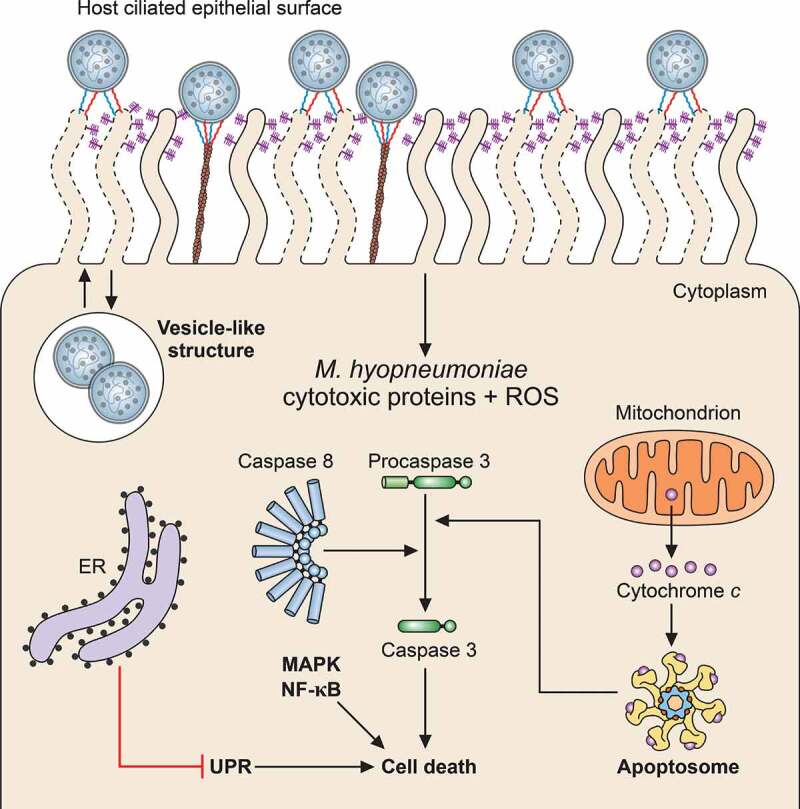
**Schematic representation of host cell damage pathways induced by *M. hyopneumoniae***. *M. hyopneumoniae* cells interact with host ciliated epithelium causing cell damage through surface-displayed cytotoxic proteins and ROS release. These cytotoxic molecules can induce host cell death by triggering different pathways, including the apoptosis cascades associated with cytochrome C, caspase-3 and −8 (apoptosome), UPR, MAPK and NF-κB. Internalization of *M. hyopneumoniae* into a vesicle-like structure in the host cell cytosol and trafficking between the intra- and extracellular milieus are also represented. The representation of *M. hyopneumoniae* cells attached to the ciliated respiratory epithelium is described in Figure 1. ER, endoplasmic reticulum

As discussed in Host immune response and immunomodulation during *M. hyopneumoniae* infection, the cytotoxicity of proteins displayed at the *M. hyopneumoniae* cell surface has been associated with immunosuppression because, at least in some cases, these proteins induce apoptosis in host immune cells. In addition, they have also been associated with epithelial cell death, causing physical damage to the swine respiratory epithelium (Figure 3), which may contribute to the lung lesions observed in swine with EP. For instance, *M. hyopneumoniae* LAMPs induce apoptosis in porcine lung epithelial cell line by activating caspase 3, caspase 8, cytochrome C, Bax, and p38 MAPK pathways [22]. Besides LAMPs, the MnuA nuclease and a putative type I signal peptidase (SPase I) from *M. hyopneumoniae* also showed pro-apoptotic effects on porcine epithelial cells [11,14]. MnuA is also secreted and, extracellularly, contributes to both NETs degradation and to host immunomodulation (as discussed in Host immune response and immunomodulation during *M. hyopneumoniae* infection). The putative SPase I, in turn, is likely involved in the secretion of many proteins, including at least some adhesins, to the cell surface or extracellular milieu (as discussed in Protein secretion of *M. hyopneumoniae* and its impact on pathogen-host interactions). The additional roles of *M. hyopneumoniae* MnuA and SPase I in *M. hyopneumoniae* biology may be the result of the specific evolutionary history of Mollicutes, which might have led to the acquisition of additional functions by some proteins to compensate in part for the observed genome reduction events [159].

Mycoplasmas can also produce ROS, by which they can induce cell death and tissue damage [160,161]. In line with that, it was demonstrated that *M. hyopneumoniae* can produce hydrogen peroxide (H_2_O_2_) when glycerol is available as a carbon source, while *M. flocculare* is unable to produce detectable amounts of this toxic molecule [162]. Notably, the gene encoding glycerol-3-phosphate oxidase (*glpO*), one of the enzymes responsible for H_2_O_2_ production in other mycoplasmas, is present in the genomes of multiple *M. hyopneumoniae* strains, but is absent in the *M. flocculare* genome [72]. However, the exact mechanism of H_2_O_2_ production in *M. hyopneumoniae* strains is still unknown. Moreover, the production of H_2_O_2_ by *M. hyopneumoniae* has the potential to cause oxidative stress to host cells and, at least *in vitro*, was associated with the antioxidant response elicited in swine respiratory epithelial cells [163].

*In vitro* experimental infections of a swine respiratory epithelial cell line with *M. hyopneumoniae* have also shown that several genes related to ciliary motility, ciliogenesis and ciliary polarization were down-regulated in infected host cells [163]. This evidence suggests a scenario in which *M. hyopneumoniae* infection could modulate the expression of genes related to the mucociliary apparatus, explaining, at least in part, the ciliostasis and loss of cilia observed in infected host tissues.

Besides the cilia damage, *M. hyopneumoniae* infection causes apoptosis-related cell events involving endoplasmic reticulum (ER) stress (Figure 3). Proteomic analyses of a tracheal swine cell line infected with *M. hyopneumoniae* revealed that infection induces dysregulation of Ca^2+^ homeostasis and ER stress [25]. It was also shown that ER stress leads to activation of the cytoprotective unfolded protein response (UPR), which, despite triggering signal transduction events associated with host defenses, is also associated with host cell apoptosis and, consequently, with damage to the ciliated respiratory epithelium. On the other hand, it was demonstrated that *M. hyopneumoniae* infection could also suppress one of the UPR pathways (the NF-κB pathway), counteracting at least in part the pro-apoptotic effects and favoring the survival of infected cells [164]. These apparently antagonistic effects of *M. hyopneumoniae* infection on UPR may represent alternative mechanisms favorable to the pathogen at different times of infection. An anti-apoptotic effect may be interesting in the initial steps of infection, to allow bacterial adherence and colonization of the host respiratory tract. The pro-apoptotic effect would be more prominent later on, contributing to tissue damage.

Additional evidences of swine epithelial cell death triggered by *M. hyopneumoniae* infection were also provided by secretome analyses of cells infected *in vitro* with pathogenic and nonpathogenic strains and with *M. flocculare* [26]. Cell death-related proteins were detected only in culture supernatants of swine cells infected with a *M. hyopneumoniae* pathogenic strain, suggesting a specific host response to pathogenic *M. hyopneumoniae*. Among these secreted cell death-related proteins, there were danger-associated molecule patterns (DAMPs), known to be secreted by host cells undergoing apoptosis during pathogen infections to alert the immune system and trigger a pro-inflammatory immune response.

## *M. hyopneumoniae* homeostasis maintenance during host colonization

The persistence of *M. hyopneumoniae* infection relies not only on its ability to modulate and evade the host immune response, but also on its arsenal of protective mechanisms to cope with different stress conditions imposed by the swine host. Moreover, *M. hyopneumoniae* survival also depends on the uptake of nutrients provided by host cells. In this final section, these aspects, which are essential for *M. hyopneumoniae* cell homeostasis, will be presented.

As discussed in Host immune response and immunomodulation during *M. hyopneumoniae* infection and Cell damage induced by *M. hyopneumoniae* infection, *M. hyopneumoniae* infection is marked by the production of ROS by both host and mycoplasma cells. However, it is not yet clear how this pathogen can protect itself from endogenously and host produced toxic metabolites. The *M. hyopneumoniae* anti-toxic metabolites arsenal is limited, as its genome lacks genes encoding important antioxidant proteins [19,72]. Thus far, four antioxidant enzymes have been identified, namely thioredoxin, thioredoxin reductase, NADH-oxidase and peroxiredoxin, and all of them were overrepresented in the *M. hyopneumoniae* 7448 pathogenic strain in comparison to the nonpathogenic strain J and *M. flocculare* [75]. This evidence provided a clear link between protection against oxidative stress and *M. hyopneumoniae* pathogenicity. Furthermore, the *M. hyopneumoniae* peroxiredoxin (MhPrx) was functionally characterized and shown to protect DNA from ROS-mediated damage *in vitro* [12,165]. Therefore, MhPrx may play an essential role in pathogen survival.

In functional “omics” studies, virtually no changes in transcript or protein levels were observed for *M. hyopneumoniae* genes/proteins classically involved in oxidative stress between stress and control conditions [166–168]. At the proteome level, it was observed that three of the four known *M. hyopneumoniae* proteins involved in the oxidative stress response (thioredoxin, NADH-oxidase and MhPrx) were among the top 20 most abundant proteins, even in the absence of oxidative stress conditions [167]. Altogether, these transcriptomic and proteomic results suggest that the anti-oxidative stress arsenal of *M. hyopneumoniae* is constitutively expressed, enabling the pathogen being always prompt to respond to this kind of stress. Interestingly, pathogenic and nonpathogenic strains of *M. hyopneumoniae* presented similar abundances of antioxidant proteins under oxidative stress [167]. On the other hand, the protein repertoire of *M. hyopneumoniae* pathogenic strain 7448 was enriched with potential virulence factors, as adhesins, nucleases and lipoproteins, upon exposure to oxidative stress in comparison to control conditions (absence of oxidative stress) and to the *M. hyopneumoniae* nonpathogenic J strain.

*Mycoplasma hyopneumoniae* must also cope with temperature shifts in the host environment due to the release of pyrogenic cytokines as a mechanism of the immune response [48,132]. A transcriptomic study regarding the expression of heat stress-related genes demonstrated that several genes, including those coding for the heat shock proteins DnaK, DnaJ, Lon proteases, and ATP-dependent serine proteinase were affected by temperature shifts [166]. However, quantitative proteomic analyses did not reveal significant differences in the abundance of these proteins between heat stress and control conditions [167]. As discussed above for the response to oxidative stress, in temperature stress conditions, the heat stress-related proteins were found to be among the most abundant, while some potential virulence factors were more abundant in the *M. hyopneumoniae* pathogenic 7448 strain under heat stress.

Taken together, the transcriptomic and the proteomic findings suggest that *M. hyopneumoniae* may require the constitutive presence of proteins involved with stress protection. These findings also suggest that the synthesis of at least some virulence factors, including adhesins, nucleases, and lipoproteins, can be triggered by oxidative and temperature stresses, which would imply increased virulence in infection (stress) conditions. Further studies will be required to improve our understanding of this relationship between stress response and virulence, and also to identify, among the non-annotated genes of *M. hyopneumoniae*, those possibly encoding so far unknown stress-response proteins.

Along with the *M. hyopneumoniae* capacity to respond to stress conditions, its metabolic capacity is essential for survival in the host environment and may contribute to virulence. The *M. hyopneumoniae* transcription unit coding for myo-inositol catabolism proteins provides a good example of that (and thus far the only one characterized regarding this aspect). *In silico* metabolic reconstructions for swine respiratory mycoplasmas demonstrated that the metabolic capacities of *M. hyopneumoniae, M. hyorhinis* and *M. flocculare* are similar [73]. However, among mycoplasma species, *M. hyopneumoniae* is the only one with a transcription unit coding for myo-inositol catabolism proteins in its genome [19,73], suggesting that it can use inositol as an alternative carbon source [169]. Indeed, it was demonstrated that *M. hyopneumoniae* strains uptake myo-inositol from culture medium, while *M. hyorhinis* and *M. flocculare* are unable to do so [162]. This capacity to uptake and metabolize the myo-inositol from the host system would allow *M. hyopneumoniae* to persist longer than *M. hyorhinis* and *M. flocculare* in the host respiratory tract [176,177].

## Conclusion

**Figure 4.**
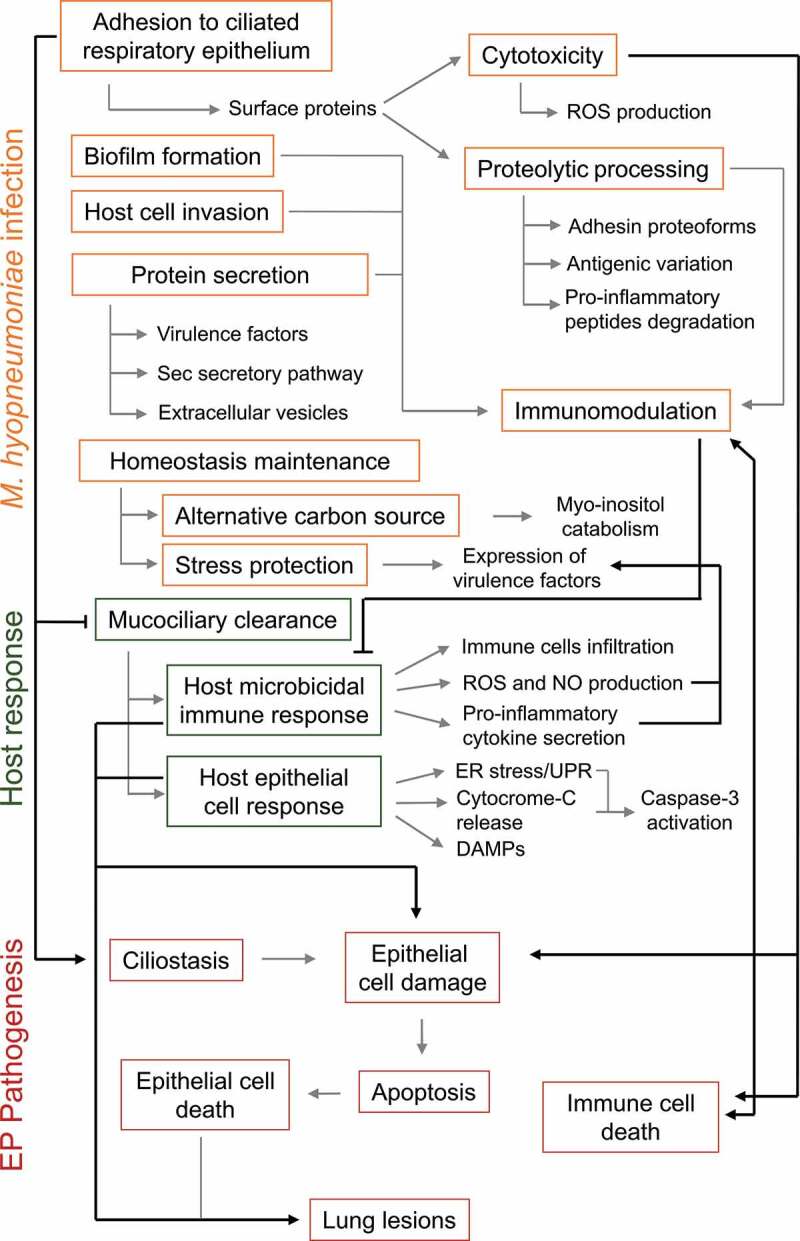
**Schematic representation of *M. hyopneumoniae* pathogenicity determinants and host responses involved in EP establishment**. Infection success depends on *M. hyopneumoniae* capacity to escape from the host mucociliary clearance and to adhere to ciliated cells of the porcine respiratory epithelium. Upon adhesion, *M. hyopneumoniae* causes ciliostasis and may form biofilms or invade host epithelial cells. *M. hyopneumoniae* also has a cytotoxic effect on host cells, causing cell damage and epithelial and immune cell death by apoptosis. This activity contributes to the modulation of the induced host immunological responses and may lead to lung lesions. Cell damage and epithelial cell death is also an outcome of host microbicidal and epithelial response to *M. hyopneumoniae* infection. Immunomodulation may also be associated with bacterial antigenic variation generated by proteolytic processing of surface adhesins and other surface proteins, and with the secretion of several virulence factors, as well as host pro-inflammatory peptides. During infection, *M. hyopneumoniae* may maintain its homeostasis using myo-inositol from lung tissue as an alternative carbon source. *M. hyopneumoniae* must also deal with stressing conditions, and its exposure to the host environment triggers the expression of several virulence factors. *M. hyopneumoniae* pathogenicity mechanisms, host response mechanisms and EP pathogenesis outcomes are shown in orange, green and red rectangles, respectively. Molecular processes and molecules involved in each mechanism or outcome are pointed out by gray arrows. Associations among *M. hyopneumoniae* pathogenicity mechanisms, host responses and EP pathogenesis outcomes are pointed out by black arrows

Regardless of its inherent simplicity, as a small, wall-less bacterium with reduced genome, *M. hyopneumoniae* utilizes several pathogenicity mechanisms, some of which are not fully understood. These mechanisms include the adherence to host ciliated epithelial cells, modulation of immune response and other host defenses, and induction of cell damage by both pathogen and host components. Moreover, comparative “omics” studies between *M. hyopneumoniae* and a nonpathogenic closely related species, *M. flocculare*, have provided evidence of proteins and functions associated with pathogenicity, as the production of differential adhesin proteoforms presented in the cell surface, the secretion of differential repertoires of proteins, and the overrepresentation of proteases, antioxidant proteins, and adhesion-related proteins in pathogenic *M. hyopneumoniae* strains. A schematic representation of the *M. hyopneumoniae* pathogenicity determinants discussed in this review is presented in Figure 4. In this complex scenario of pathogen-host interplay, more studies are needed to corroborate the available evidence and to elucidate further aspects of *M. hyopneumoniae* pathogenicity, especially those related to the *in vivo* infection process. Overall, the identification of *M. hyopneumoniae* pathogenicity determinants and their mechanisms of action are of utmost relevance to discover new and efficient targets for the development of novel diagnostic methods, therapeutic drugs, and preventive vaccines against EP.

## Data Availability

Data sharing is not applicable to this article as no new data were created or analyzed in this study.

## References

[1] Thacker EL, Minion CF. Mycoplasmosis. In: Jeffrey J. Zimmerman, Locke A. Karriker, Alejandro Ramirez, Ken J. Schwartz and Gregory W. Stevenson, editors. Diseases of swine. Iowa state: university press; 2010. p. 779–797.

[2] Maes D, Sibila M, Kuhnert P, et al. Update on mycoplasma hyopneumoniae infections in pigs: knowledge gaps for improved disease control. Transbound Emerg Dis. 2018;65(1):110-124. DOI:10.1111/tbed.12677 28834294

[3] Holst S, Yeske P, Pieters M. Elimination of mycoplasma hyopneumoniae from breed-to-wean farms: a review of current protocols with emphasis on herd closure and medication. J Swine Health Prod. 2015;23(6):321–330.

[4] Bai Y, Gan Y, Hua LZ, et al. Application of a sIgA-ELISA method for differentiation of Mycoplasma hyopneumoniae infected from vaccinated pigs. Vet Microbiol. 2018;223:86–92. 30173757 10.1016/j.vetmic.2018.07.023

[5] Feng ZX, Bai Y, Yao JT, et al. Use of serological and mucosal immune responses to Mycoplasma hyopneumoniae antigens P97R1, P46 and P36 in the diagnosis of infection. Vet J. 2014;202:128–133. 25066030 10.1016/j.tvjl.2014.06.019

[6] Galli V, Simionatto S, Marchioro SB, et al. Immunisation of mice with Mycoplasma hyopneumoniae antigens P37, P42, P46 and P95 delivered as recombinant subunit or DNA vaccines. Vaccine. 2012;31(1):135–140. 23137841 10.1016/j.vaccine.2012.10.088

[7] Marchioro SB, Fisch A, Gomes CK, et al. Local and systemic immune responses induced by a recombinant chimeric protein containing Mycoplasma hyopneumoniae antigens fused to the B subunit of Escherichia coli heat-labile enterotoxin LTB. Vet Microbiol. 2014;173(1–2):166–171. 25091529 10.1016/j.vetmic.2014.07.009

[8] Virginio VG, Gonchoroski T, Paes JA, et al. Immune responses elicited by Mycoplasma hyopneumoniae recombinant antigens and DNA constructs with potential for use in vaccination against porcine enzootic pneumonia. Vaccine. 2014;32(44):5832–5838. 25148775 10.1016/j.vaccine.2014.08.008

[9] Jarocki VM, Raymond BBA, Tacchi JL, et al. Mycoplasma hyopneumoniae surface-associated proteases cleave bradykinin, substance P, neurokinin A and neuropeptide Y. Sci Rep. 2019;9:14585. 31601981 10.1038/s41598-019-51116-w PMC6787215

[10] Jarocki VM, Santos J, Tacchi JL, et al. MHJ_0461 is a multifunctional leucine aminopeptidase on the surface of Mycoplasma hyopneumoniae. Open Biol. 2015;5:140175. 25589579 10.1098/rsob.140175 PMC4313372

[11] Li P, Zhang Y, Li X, et al. Mycoplasma hyopneumoniae Mhp597 is a cytotoxicity, inflammation and immunosuppression associated nuclease. Vet Microbiol. 2019;235:53–62. 31282379 10.1016/j.vetmic.2019.05.011

[12] Machado C, Pinto P, Zaha A, et al. A peroxiredoxin from Mycoplasma hyopneumoniae with a possible role in H2O2 detoxification. Microbiology. 2009;155(10):3411–3419. 19589831 10.1099/mic.0.030643-0

[13] Moitinho-Silva L, Heineck BL, Reolon LA, et al. Mycoplasma hyopneumoniae type I signal peptidase: expression and evaluation of its diagnostic potential. Vet Microbiol. 2012;154(3–4):282–291. 21831542 10.1016/j.vetmic.2011.07.009

[14] Paes JA, Virginio VG, Cancela M, et al. Pro-apoptotic effect of a Mycoplasma hyopneumoniae putative type I signal peptidase on PK(15) swine cells. Vet Microbiol. 2017b;201:170–176. 28284605 10.1016/j.vetmic.2017.01.024

[15] Tacchi JL, Raymond BB, Jarocki VM, et al. Cilium adhesin P216 (MHJ_0493) is a target of ectodomain shedding and aminopeptidase activity on the surface of Mycoplasma hyopneumoniae. J Proteome Res. 2014;13:2920–2930. 24804907 10.1021/pr500087c

[16] Pirofski LA, Casadevall A. The damage-response framework as a tool for the physician-scientist to understand the pathogenesis of infectious diseases. J Infect Dis. 2018;218:S7–S11. 30124977 10.1093/infdis/jiy083 PMC6093430

[17] Mare CJ, Switzer WP. New species: mycoplasma hyopneumoniae; a causative agent of virus pig pneumonia. Vet Med Small Anim Clin. 1965;60:841–846. 14323369

[18] Minion F, Lefkowitz E, Madsen M, et al. The genome sequence of Mycoplasma hyopneumoniae strain 232, the agent of swine mycoplasmosis. J Bacteriol. 2004;186(21):7123–7133. 15489423 10.1128/JB.186.21.7123-7133.2004 PMC523201

[19] Vasconcelos AT, Ferreira HB, Bizarro CV, et al. Swine and poultry pathogens: the complete genome sequences of two strains of Mycoplasma hyopneumoniae and a strain of Mycoplasma synoviae. J Bacteriol. 2005;187:5568–5577. 16077101 10.1128/JB.187.16.5568-5577.2005 PMC1196056

[20] Zielinski GC, Ross RF. Effect of growth in cell cultures and strain on virulence of mycoplasma hyopneumoniae for swine. Am J Vet Res. 1990;51:344–348. 2180349

[21] Bai F, Ni B, Liu M, et al. Mycoplasma hyopneumoniae-derived lipid-associated membrane proteins induce apoptosis in porcine alveolar macrophage via increasing nitric oxide production, oxidative stress, and caspase-3 activation. Vet Immunol Immunopathol. 2013;155(3):155–161. 23928261 10.1016/j.vetimm.2013.07.004

[22] Ni B, Bai FF, Wei Y, et al. Apoptosis induced by lipid-associated membrane proteins from Mycoplasma hyopneumoniae in a porcine lung epithelial cell line with the involvement of caspase 3 and the MAPK pathway. Genet Mol Res. 2015;14(3):11429–11443. 26436384 10.4238/2015.September.25.10

[23] Ni L, Song C, Wu X, et al. RNA-seq transcriptome profiling of porcine lung from two pig breeds in response to Mycoplasma hyopneumoniae infection. PeerJ. 2019;7:e7900. 31656701 10.7717/peerj.7900 PMC6812673

[24] Trueeb BS, Braun RO, Auray G, et al. Differential innate immune responses induced by Mycoplasma hyopneumoniae and Mycoplasma hyorhinis in various types of antigen presenting cells. Vet Microbiol. 2020;240:108541. 31902489 10.1016/j.vetmic.2019.108541

[25] Leal Zimmer FMA, Moura H, Barr JR, et al. Intracellular changes of a swine tracheal cell line infected with a Mycoplasma hyopneumoniae pathogenic strain. Microb Pathog. 2019a;137:103717. 31494300 10.1016/j.micpath.2019.103717

[26] Leal Zimmer FMDA, Paludo GP, Moura H, et al. Differential secretome profiling of a swine tracheal cell line infected with mycoplasmas of the swine respiratory tract. J Proteomics. 2019b;192:147–159. 30176387 10.1016/j.jprot.2018.08.018

[27] Maes D, Segales J, Meyns T, et al. Control of Mycoplasma hyopneumoniae infections in pigs. Vet Microbiol. 2008;126(4):297–309. 17964089 10.1016/j.vetmic.2007.09.008 PMC7130725

[28] He Y, Xu MJ, Zhou DH, et al. Seroprevalence of Mycoplasma hyopneumoniae in pigs in subtropical southern China. Trop Anim Health Prod. 2011;43(3):695–698. 21107906 10.1007/s11250-010-9755-3

[29] Sosa C, Blois A, Ibáñez F, et al. Genetic diversity of mycoplasma hyopneumoniae in Mmendoza province. Rev Argent Microbiol. 2019;51:229–233. 30651187 10.1016/j.ram.2018.07.004

[30] Pacce VD, Oliveira NRD, Jorge S, et al. Occurrence of mycoplasma hyopneumoniae in slaughter pigs from Southern Brazil. BrazilJ Veterinary Res Animal Sci. 2019;5:6.

[31] Vicente AF, Catto D, Allendorf SD, et al. Seropositivity for *mycoplasma hyopneumoniae* in pigs at a slaughterhouse in the central region of São Paulo. Arquivo Brasileiro De Medicina Veterinária E Zootecnia. 2013;65:5.

[32] Oba P, Wieland B, Mwiine FN, et al. Status and gaps of research on respiratory disease pathogens of swine in Africa. Porcine Health Manag. 2020;6:5. 32257367 10.1186/s40813-020-0144-7 PMC7066813

[33] Bertelloni F, Mazzei M, Cilia G, et al. Serological Survey on Bacterial and Viral Pathogens in Wild Boars Hunted in Tuscany. Ecohealth. 2020;17(1):85–93. 32034585 10.1007/s10393-020-01475-y

[34] Malmsten A, Magnusson U, Ruiz-Fons F, et al. A serologic survey of pathogens in wild boar (sus scrofa) in sweden. J Wildl Dis. 2018;54:229–237. 29377751 10.7589/2017-05-120

[35] Calsamiglia M, Pijoan C. Colonisation state and colostral immunity to Mycoplasma hyopneumoniae of different parity sows. Vet Rec. 2000;146(18):530–532. 11321216 10.1136/vr.146.18.530

[36] Nathues H, Doehring S, Woeste H, et al. Individual risk factors for Mycoplasma hyopneumoniae infections in suckling pigs at the age of weaning. Acta Vet Scand. 2013;55:44. 23731650 10.1186/1751-0147-55-44 PMC3698135

[37] Fano E, Pijoan C, Dee S, et al. Effect of mycoplasma hyopneumoniae colonization at weaning on disease severity in growing pigs. Can J Vet Res. 2007;71:195–200. 17695594 PMC1899865

[38] Sibila M, Nofrarías M, López-Soria S, et al. Chronological study of Mycoplasma hyopneumoniae infection, seroconversion and associated lung lesions in vaccinated and non-vaccinated pigs. Vet Microbiol. 2007;122:97–107. 17303351 10.1016/j.vetmic.2007.01.010

[39] Meyns T, Maes D, Dewulf J, et al. Quantification of the spread of Mycoplasma hyopneumoniae in nursery pigs using transmission experiments. Prev Vet Med. 2004;66(1–4):265–275. 15579347 10.1016/j.prevetmed.2004.10.001

[40] Roos LR, Fano E, Homwong N, et al. A model to investigate the optimal seeder-to-naïve ratio for successful natural Mycoplasma hyopneumoniae gilt exposure prior to entering the breeding herd. Vet Microbiol. 2016;184:51–58. 26854344 10.1016/j.vetmic.2016.01.008

[41] Villarreal I, Meyns T, Dewulf J, et al. The effect of vaccination on the transmission of Mycoplasma hyopneumoniae in pigs under field conditions. Vet J. 2011;188(1):48–52. 20605734 10.1016/j.tvjl.2010.04.024

[42] Pieters M, Pijoan C, Fano E, et al. An assessment of the duration of Mycoplasma hyopneumoniae infection in an experimentally infected population of pigs. Vet Microbiol. 2009;134(3–4):261–266. 18835112 10.1016/j.vetmic.2008.08.016

[43] Takeuti KL, de Barcellos DESN, de Lara AC, et al. Detection of Mycoplasma hyopneumoniae in naturally infected gilts over time. Vet Microbiol. 2017;203:215–220. 28619147 10.1016/j.vetmic.2017.03.025

[44] Mayor D, Zeeh F, Frey J, et al. Diversity of Mycoplasma hyopneumoniae in pig farms revealed by direct molecular typing of clinical material. Vet Res. 2007;38(3):391–398. 17506969 10.1051/vetres:2007006

[45] Mayor D, Jores J, Korczak BM, et al. Multilocus sequence typing (MLST) of Mycoplasma hyopneumoniae: a diverse pathogen with limited clonality. Vet Microbiol. 2008;127(1–2):63–72. 17884308 10.1016/j.vetmic.2007.08.010

[46] de Castro LA, Rodrigues Pedroso T, Kuchiishi SS, et al. Variable number of tandem aminoacid repeats in adhesion-related CDS products in Mycoplasma hyopneumoniae strains. Vet Microbiol. 2006;116:258–269. 16730926 10.1016/j.vetmic.2006.04.022

[47] Dos Santos LF, Sreevatsan S, Torremorell M, et al. Genotype distribution of Mycoplasma hyopneumoniae in swine herds from different geographical regions. Vet Microbiol. 2015;175:374–381. 25497236 10.1016/j.vetmic.2014.11.018

[48] Hwang, M.H., Damte, D., Lee, J.S., Gebru, E., Chang, Z.Q., Cheng, H., Jung, B.Y., Rhee, M.H., Park, S.C. 2011. Mycoplasma hyopneumoniae induces proinflammatory cytokine and nitric oxide production through NFκB and MAPK pathways in RAW264.7 cells. Vet Res Commun 35, 21–34. 10.1007/s11259-010-9447-5 21104123

[49] Garza-Moreno L, Segalés J, Pieters M, et al. Acclimation strategies in gilts to control Mycoplasma hyopneumoniae infection. Vet Microbiol. 2018;219:23–29. 29778201 10.1016/j.vetmic.2018.04.005

[50] Betlach AM, Maes D, Garza-Moreno L, et al. Mycoplasma hyopneumoniae variability: current trends and proposed terminology for genomic classification. Transbound Emerg Dis. 2019. doi:10.1111/tbed.13233 31099490

[51] Vranckx K, Maes D, Sacristán REP, et al. A longitudinal study of the diversity and dynamics of Mycoplasma hyopneumoniae infections in pig herds. Vet Microbiol. 2012;156:315–321. 22138620 10.1016/j.vetmic.2011.11.007

[52] Tao Y, Shu J, Chen J, et al. A concise review of vaccines against Mycoplasma hyopneumoniae. Res Vet Sci. 2019;123:144–152. 30665029 10.1016/j.rvsc.2019.01.007

[53] Meyns T, Dewulf J, de Kruif A, et al. Comparison of transmission of Mycoplasma hyopneumoniae in vaccinated and non-vaccinated populations. Vaccine. 2006;24(49–50):7081–7086. 16934376 10.1016/j.vaccine.2006.07.004

[54] Villarreal I, Vranckx K, Calus D, et al. Effect of challenge of pigs previously immunised with inactivated vaccines containing homologous and heterologous Mycoplasma hyopneumoniae strains. BMC Vet Res. 2012;8(1):2. 22225838 10.1186/1746-6148-8-2 PMC3269371

[55] Beffort L, Weiß C, Fiebig K, et al. Field study on the safety and efficacy of intradermal versus intramuscular vaccination against Mycoplasma hyopneumoniae. Vet Rec. 2017;181(13):348. 28893974 10.1136/vr.104466

[56] Maes D, Sibila M, Kuhnert P, et al. Update on Mycoplasma hyopneumoniae infections in pigs: knowledge gaps for improved disease control. Transbound Emerg Dis. 2018;65(Suppl 1):110–124. 28834294 10.1111/tbed.12677

[57] Chae C. Porcine respiratory disease complex:iInteraction of vaccination and porcine circovirus type 2, porcine reproductive and respiratory syndrome virus, and Mycoplasma hyopneumoniae. Vet J. 2016;212:1–6. 27256017 10.1016/j.tvjl.2015.10.030

[58] Feng ZX, Wei YN, Li GL, et al. Development and validation of an attenuated Mycoplasma hyopneumoniae aerosol vaccine. Vet Microbiol. 2013;167:417–424. 24035264 10.1016/j.vetmic.2013.08.012

[59] Martelli P, Terreni M, Guazzetti S, et al. Antibody response to Mycoplasma hyopneumoniae infection in vaccinated pigs with or without maternal antibodies induced by sow vaccination. J Vet Med B Infect Dis Vet Public Health. 2006;53(5):229–233. 16732881 10.1111/j.1439-0450.2006.00952.x

[60] Pieters M, Fano E, Pijoan C, et al. An experimental model to evaluate mycoplasma hyopneumoniae transmission from asymptomatic carriers to unvaccinated and vaccinated sentinel pigs. Can J Vet Res. 2010;74:157–160. 20592848 PMC2851728

[61] de Oliveira NR, Jorge S, Gomes CK, et al. A novel chimeric protein composed of recombinant Mycoplasma hyopneumoniae antigens as a vaccine candidate evaluated in mice. Vet Microbiol. 2017;201:146–153. 28284602 10.1016/j.vetmic.2017.01.023

[62] Jorge S, de Oliveira NR, Marchioro SB, et al. The Mycoplasma hyopneumoniae recombinant heat shock protein P42 induces an immune response in pigs under field conditions. Comp Immunol Microbiol Infect Dis. 2014;37(4):229–236. 25082621 10.1016/j.cimid.2014.07.001

[63] Tassis PD, Tsakmakidis I, Papatsiros VG, et al. A randomized controlled study on the efficacy of a novel combination vaccine against enzootic pneumonia (Mycoplasma hyopneumoniae) and porcine Circovirus type 2 (PCV2) in the presence of strong maternally derived PCV2 immunity in pigs. BMC Vet Res. 2017;13(1):91. 28388953 10.1186/s12917-017-1014-7 PMC5384188

[64] Virginio VG, Bandeira NC, Leal FM, et al. Assessment of the adjuvant activity of mesoporous silica nanoparticles in recombinant. Heliyon. 2017;3:e00225. 28194450 10.1016/j.heliyon.2016.e00225 PMC5291748

[65] Drexler CS, Witvliet MH, Raes M, et al. Efficacy of combined porcine reproductive and respiratory syndrome virus and Mycoplasma hyopneumoniae vaccination in piglets. Vet Rec. 2010;166(3):70–74. 20081177 10.1136/vr.c176

[66] Jeong J, Park C, Choi K, et al. A new single-dose bivalent vaccine of porcine circovirus type 2 and Mycoplasma hyopneumoniae elicits protective immunity and improves growth performance under field conditions. Vet Microbiol. 2016;182:178–186. 26711046 10.1016/j.vetmic.2015.11.023

[67] Park C, Jeong J, Choi K, et al. Efficacy of a new bivalent vaccine of porcine circovirus type 2 and Mycoplasma hyopneumoniae (Fostera™ PCV MH) under experimental conditions. Vaccine. 2016;34(2):270–275. 26626212 10.1016/j.vaccine.2015.11.034

[68] Roques E, Girard A, Gagnon CA, et al. Antibody responses induced in mice immunized with recombinant adenovectors expressing chimeric proteins of various porcine pathogens. Vaccine. 2013;31(24):2698–2704. 23583895 10.1016/j.vaccine.2013.03.068

[69] Duivon D, Corrégé I, Hémonic A, et al. Field evaluation of piglet vaccination with a. Porcine Health Manag. 2018;4:4. 29375890 10.1186/s40813-017-0077-y PMC5772722

[70] Maes D, Boyen F, Haesebrouck F, et al. Antimicrobial treatment of Mycoplasma hyopneumoniae infections. Vet J. 2020;259-260:105474. 32553237 10.1016/j.tvjl.2020.105474

[71] Vicca J, Stakenborg T, Maes D, et al. In vitro susceptibilities of Mycoplasma hyopneumoniae field isolates. Antimicrob Agents Chemother. 2004;48(11):4470–4472. 15504886 10.1128/AAC.48.11.4470-4472.2004 PMC525426

[72] Felde O, Kreizinger Z, Sulyok KM, et al. Antibiotic susceptibility testing of Mycoplasma hyopneumoniae field isolates from Central Europe for fifteen antibiotics by microbroth dilution method. PLoS One. 2018;13(12):e0209030. 30533041 10.1371/journal.pone.0209030 PMC6289410

[73] Qiu G, Rui Y, Zhang J, et al. Macrolide-resistance selection in Tibetan pigs with a high load of Mycoplasma hyopneumoniae. Microb Drug Resist. 2018;24(7):1043–1049. 29271698 10.1089/mdr.2017.0254

[74] Siqueira FM, Thompson CE, Virginio VG, et al. New insights on the biology of swine respiratory tract mycoplasmas from a comparative genome analysis. BMC Genomics. 2013;14:175. 23497205 10.1186/1471-2164-14-175 PMC3610235

[75] Ferrarini MG, Siqueira FM, Mucha SG, et al. Insights on the virulence of swine respiratory tract mycoplasmas through genome-scale metabolic modeling. BMC Genomics. 2016;17:353. 27178561 10.1186/s12864-016-2644-z PMC4866288

[76] Paes JA, Lorenzatto KR, de Moraes SN, et al. Secretomes of Mycoplasma hyopneumoniae and Mycoplasma flocculare reveal differences associated to pathogenesis. J Proteomics. 2017a;154:69–77. 28003119 10.1016/j.jprot.2016.12.002

[77] Paes JA, Machado LDPN, Dos Anjos Leal FM, et al. Comparative proteomics of two Mycoplasma hyopneumoniae strains and Mycoplasma flocculare identified potential porcine enzootic pneumonia determinants. Virulence. 2018;9:1230–1246. 30027802 10.1080/21505594.2018.1499379 PMC6104684

[78] Siqueira FM, Gerber AL, Guedes RL, et al. Unravelling the transcriptome profile of the Swine respiratory tract mycoplasmas. PLoS One. 2014;9:e110327. 25333523 10.1371/journal.pone.0110327 PMC4198240

[79] Raymond BBA, Madhkoor R, Schleicher I, et al. Extracellular Actin Is a Receptor for. Front Cell Infect Microbiol. 2018b;8:54. 29535975 10.3389/fcimb.2018.00054 PMC5835332

[80] Tacchi JL, Raymond BB, Haynes PA, et al. Post-translational processing targets functionally diverse proteins in mycoplasma hyopneumoniae. Open Biol. 2016;6(2):150210. 10.1098/rsob.150210 PMC4772806 26865024

[81] Hammerschmidt S, Rohde M, Preer KT. Extracellular matrix interactions with gram-positive pathogens. Microbiol Spectr. 2019;7(2). 10.1128/microbiolspec.gpp3-0041-2018 PMC11590433 31004421

[82] Bustamante-Marin XM, Ostrowski LE. Cilia and mucociliary clearance. Cold Spring Harb Perspect Biol. 2017;9(4):a028241. 10.1101/cshperspect.a028241 PMC5378048 27864314

[83] Blanchard B, Vena M, Cavalier A, et al. Electron microscopic observation of the respiratory tract of SPF piglets inoculated with Mycoplasma hyopneumoniae. Vet Microbiol. 1992;30(4):329–341. 1533978 10.1016/0378-1135(92)90020-t

[84] DeBey MC, Ross RF. Ciliostasis and loss of cilia induced by Mycoplasma hyopneumoniae in porcine tracheal organ cultures. Infect Immun. 1994;62:5312–5318. 7960110 10.1128/iai.62.12.5312-5318.1994 PMC303270

[85] Wang H, Zhang Z, Xie X, et al. Paracellular pathway-mediated Mycoplasma hyopneumoniae Migration across Porcine Airway Epithelial Barrier under Air-Liquid Interface Conditions. Infect Immun. 2020;88(10):e00470-20. 10.1128/IAI.00470-20 PMC7504950 32747599

[86] Chen R, Yu Y, Feng Z, et al. Featured species-specific loops are found in the crystal structure of Mhp Eno, a Cell Surface Adhesin From Mycoplasma hyopneumoniae. Front Cell Infect Microbiol. 2019;9:209. 31263685 10.3389/fcimb.2019.00209 PMC6585157

[87] Yu Y, Liu M, Hua L, et al. Fructose-1,6-bisphosphate aldolase encoded by a core gene of Mycoplasma hyopneumoniae contributes to host cell adhesion. Vet Res. 2018a;49(1):114. 30454073 10.1186/s13567-018-0610-2 PMC6245935

[88] Reolon LA, Martello CL, Schrank IS, et al. Survey of surface proteins from the pathogenic Mycoplasma hyopneumoniae strain 7448 using a biotin cell surface labeling approach. PLoS One. 2014;9(11):e112596. 25386928 10.1371/journal.pone.0112596 PMC4227723

[89] Young TF, Thacker EL, Erickson BZ, et al. A tissue culture system to study respiratory ciliary epithelial adherence of selected swine mycoplasmas. Vet Microbiol. 2000;71(3–4):269–279. 10703709 10.1016/s0378-1135(99)00176-5

[90] Berry IJ, Jarocki VM, Tacchi JL, et al. N-terminomics identifies widespread endoproteolysis and novel methionine excision in a genome-reduced bacterial pathogen. Sci Rep. 2017;7:11063. 28894154 10.1038/s41598-017-11296-9 PMC5593965

[91] Bogema DR, Scott NE, Padula MP, et al. Sequence TTKF ↓ QE defines the site of proteolytic cleavage in Mhp683 protein, a novel glycosaminoglycan and cilium adhesin of Mycoplasma hyopneumoniae. J Biol Chem. 2011;286:41217–41229. 21969369 10.1074/jbc.M111.226084 PMC3308835

[92] Deutscher AT, Tacchi JL, Minion FC, et al. Mycoplasma hyopneumoniae surface proteins Mhp385 and Mhp384 bind host cilia and glycosaminoglycans and are endoproteolytically processed by proteases that recognize different cleavage motifs. J Proteome Res. 2012;11(3):1924–1936. 22229926 10.1021/pr201115v

[93] Djordjevic SP, Cordwell SJ, Djordjevic MA, et al. Proteolytic processing of the Mycoplasma hyopneumoniae cilium adhesin. Infect Immun. 2004;72(5):2791–2802. 15102789 10.1128/IAI.72.5.2791-2802.2004 PMC387856

[94] Machado LDPN, Paes JA, Souza Dos Santos P, et al. Evidences of differential endoproteolytic processing on the surfaces of mycoplasma hyopneumoniae and mycoplasma flocculare. Microb Pathog. 2019;140:103958. 31899326 10.1016/j.micpath.2019.103958

[95] Raymond BB, Jenkins C, Seymour LM, et al. Proteolytic processing of the cilium adhesin MHJ_0194 (P123J) in Mycoplasma hyopneumoniae generates a functionally diverse array of cleavage fragments that bind multiple host molecules. Cell Microbiol. 2015;17:425–444. 25293691 10.1111/cmi.12377

[96] Machado LDPN, Paes JA, Souza Dos Santos P, et al. Evidences of differential endoproteolytic processing on the surfaces of Mycoplasma hyopneumoniae and Mycoplasma flocculare. Microb Pathog. 2020;140:103958. 31899326 10.1016/j.micpath.2019.103958

[97] Robinson MW, Buchtmann KA, Jenkins C, et al. MHJ_0125 is an M42 glutamyl aminopeptidase that moonlights as a multifunctional adhesin on the surface of Mycoplasma hyopneumoniae. Open Biol. 2013;3:130017. 23594879 10.1098/rsob.130017 PMC3718333

[98] Widjaja M, Harvey KL, Hagemann L, et al. Elongation factor Tu is a multifunctional and processed moonlighting protein. Sci Rep. 2017;7:11227. 28894125 10.1038/s41598-017-10644-z PMC5593925

[99] Zhang Q, Young TF, Ross RF. Glycolipid receptors for attachment of Mycoplasma hyopneumoniae to porcine respiratory ciliated cells. Infect Immun. 1994;62:4367–4373. 7927697 10.1128/iai.62.10.4367-4373.1994 PMC303118

[100] Deutscher AT, Jenkins C, Minion FC, et al. Repeat regions R1 and R2 in the P97 paralogue Mhp271 of Mycoplasma hyopneumoniae bind heparin, fibronectin and porcine cilia. Mol Microbiol. 2010;78(2):444–458. 20879998 10.1111/j.1365-2958.2010.07345.x

[101] Seymour LM, Falconer L, Deutscher AT, et al. Mhp107 is a member of the multifunctional adhesin family of Mycoplasma hyopneumoniae. J Biol Chem. 2011;286(12):10097–10104. 21245147 10.1074/jbc.M110.208140 PMC3060461

[102] Seymour LM, Jenkins C, Deutscher AT, et al. Mhp182 (P102) binds fibronectin and contributes to the recruitment of plasmin(ogen) to the Mycoplasma hyopneumoniae cell surface. Cell Microbiol. 2012;14:81–94. 21951786 10.1111/j.1462-5822.2011.01702.x

[103] Burnett TA, Dinkla K, Rohde M, et al. P159 is a proteolytically processed, surface adhesin of Mycoplasma hyopneumoniae: defined domains of P159 bind heparin and promote adherence to eukaryote cells. Mol Microbiol. 2006;60(3):669–686. 16629669 10.1111/j.1365-2958.2006.05139.x

[104] Wilton J, Jenkins C, Cordwell SJ, et al. Mhp493 (P216) is a proteolytically processed, cilium and heparin binding protein of Mycoplasma hyopneumoniae. Mol Microbiol. 2009;71(3):566–582. 19040640 10.1111/j.1365-2958.2008.06546.x

[105] Grimmer J, Dumke R. Organization of multi-binding to host proteins: the glyceraldehyde-3-phosphate dehydrogenase (GAPDH) of Mycoplasma pneumoniae. Microbiol Res. 2019;218:22–31. 30454655 10.1016/j.micres.2018.09.006

[106] Gründel A, Jacobs E, Dumke R. Interactions of surface-displayed glycolytic enzymes of Mycoplasma pneumoniae with components of the human extracellular matrix. Int J Med Microbiol. 2016;306(8):675–685. 27616280 10.1016/j.ijmm.2016.09.001

[107] Hagemann L, Gründel A, Jacobs E, et al. The surface-displayed chaperones GroEL and DnaK of mycoplasma pneumoniae interact with human plasminogen and components of the extracellular matrix. Pathog Dis. 2017;75(3). 10.1093/femspd/ftx017 28204467

[108] Huang J, Zhu H, Wang J, et al. Fructose-1,6-bisphosphate aldolase is involved in Mycoplasma bovis colonization as a fibronectin-binding adhesin. Res Vet Sci. 2019;124:70–78. 30852357 10.1016/j.rvsc.2019.02.010

[109] Qi J, Zhang F, Wang Y, et al. Characterization of Mycoplasma gallisepticum pyruvate dehydrogenase alpha and beta subunits and their roles in cytoadherence. PLoS One. 2018;13(12):e0208745. 30532176 10.1371/journal.pone.0208745 PMC6287819

[110] Raymond BBA, Turnbull L, Jenkins C, et al. Mycoplasma hyopneumoniae resides intracellularly within porcine epithelial cells. Sci Rep. 2018c;8(1):17697. 30523267 10.1038/s41598-018-36054-3 PMC6283846

[111] Marois C, Le Carrou J, Kobisch M, et al. Isolation of Mycoplasma hyopneumoniae from different sampling sites in experimentally infected and contact SPF piglets. Vet Microbiol. 2007;120(1–2):96–104. 17116374 10.1016/j.vetmic.2006.10.015

[112] Seymour LM, Deutscher AT, Jenkins C, et al. A processed multidomain Mycoplasma hyopneumoniae adhesin binds fibronectin, plasminogen, and swine respiratory cilia. J Biol Chem. 2010;285(44):33971–33978. 20813843 10.1074/jbc.M110.104463 PMC2962497

[113] Widjaja M, Berry IJ, Jarocki VM, et al. Cell surface processing of the P1 adhesin of mycoplasma pneumoniae identifies novel domains that bind host molecules. Sci Rep. 2020;10:6384. 32286369 10.1038/s41598-020-63136-y PMC7156367

[114] Bürki S, Frey J, Pilo P. Virulence, persistence and dissemination of Mycoplasma bovis. Vet Microbiol. 2015;179(1–2):15–22. 25824130 10.1016/j.vetmic.2015.02.024

[115] Chen H, Yu S, Hu M, et al. Identification of biofilm formation by Mycoplasma gallisepticum. Vet Microbiol. 2012;161(1–2):96–103. 22840542 10.1016/j.vetmic.2012.07.013

[116] Simmons WL, Daubenspeck JM, Osborne JD, et al. Type 1 and type 2 strains of Mycoplasma pneumoniae form different biofilms. Microbiology. 2013;159(Pt_4):737–747. 23412845 10.1099/mic.0.064782-0 PMC4036059

[117] Raymond BBA, Jenkins C, Turnbull L, et al. Extracellular DNA release from the genome-reduced pathogen Mycoplasma hyopneumoniae is essential for biofilm formation on abiotic surfaces. Sci Rep. 2018a;8:10373. 29991767 10.1038/s41598-018-28678-2 PMC6039474

[118] Tassew DD, Mechesso AF, Park NH, et al. Biofilm formation and determination of minimum biofilm eradication concentration of antibiotics in Mycoplasma hyopneumoniae. J Vet Med Sci. 2017;79:1716–1720. 28890520 10.1292/jvms.17-0279 PMC5658566

[119] Dallo SF, Baseman JB. Intracellular DNA replication and long-term survival of pathogenic mycoplasmas. Microb Pathog. 2000;29(5):301–309. 11031124 10.1006/mpat.2000.0395

[120] Yavlovich A, Tarshis M, Rottem S. Internalization and intracellular survival of Mycoplasma pneumoniae by non-phagocytic cells. FEMS Microbiol Lett. 2004;233(2):241–246. 15063492 10.1016/j.femsle.2004.02.016

[121] Raymond BB, Djordjevic S. Exploitation of plasmin(ogen) by bacterial pathogens of veterinary significance. Vet Microbiol. 2015;178:1–13. 25937317 10.1016/j.vetmic.2015.04.008

[122] Woolley LK, Fell SA, Djordjevic SP, et al. Plasmin activity in the porcine airways is enhanced during experimental infection with Mycoplasma hyopneumoniae, is positively correlated with proinflammatory cytokine levels and is ameliorated by vaccination. Vet Microbiol. 2013;164(1–2):60–66. 23490555 10.1016/j.vetmic.2013.02.003

[123] Bhattacharya S, Ploplis VA, Castellino FJ. Bacterial plasminogen receptors utilize host plasminogen system for effective invasion and dissemination. J Biomed Biotechnol. 2012;2012:482096. 23118509 10.1155/2012/482096 PMC3477821

[124] Sinha B, François PP, Nüsse O, et al. Fibronectin-binding protein acts as Staphylococcus aureus invasin via fibronectin bridging to integrin alpha5beta1. Cell Microbiol. 1999;1:101–117. 11207545 10.1046/j.1462-5822.1999.00011.x

[125] Friis NF. Mycoplasm suipneumoniae and mycoplasma flocculare in comparative pathogenicity studies. Acta Vet Scand. 1974;15:507–518. 4455082 10.1186/BF03547222 PMC8407235

[126] Le Carrou J, Laurentie M, Kobisch M, et al. Persistence of Mycoplasma hyopneumoniae in experimentally infected pigs after marbofloxacin treatment and detection of mutations in the parC gene. Antimicrob Agents Chemother. 2006;50(6):1959–1966. 16723552 10.1128/AAC.01527-05 PMC1479153

[127] Deeney AS, Maglennon GA, Chapat L, et al. Mycoplasma hyopneumoniae evades phagocytic uptake by porcine alveolar macrophages in vitro. Vet Res. 2019;50(1):51. 31234931 10.1186/s13567-019-0667-6 PMC6591956

[128] Green ER, Mecsas J. Bacterial secretion systems: an overview. Microbiol Spectr. 2016;4(1). 10.1128/microbiolspec.VMBF-0012-2015 PMC4804464 26999395

[129] Smets D, Loos MS, Karamanou S, et al. Protein transport across the bacterial plasma membrane by the sec pathway. Protein J. 2019;38(3):262–273. 31134461 10.1007/s10930-019-09841-8

[130] Chernov VM, Mouzykantov AA, Baranova NB, et al. Extracellular membrane vesicles secreted by mycoplasma Acholeplasma laidlawii PG8 are enriched in virulence proteins. J Proteomics. 2014;110C:117–128. 10.1016/j.jprot.2014.07.020 25088052

[131] Gaurivaud P, Ganter S, Villard A, et al. Mycoplasmas are no exception to extracellular vesicles release: revisiting old concepts. PLoS One. 2018;13(11):e0208160. 30485365 10.1371/journal.pone.0208160 PMC6261642

[132] Rodríguez F, Batista M, Hernández JN, et al. Relationship between expression of interleukin-5 and Interleukin-13 by epithelial cells and bronchiolar changes in pigs infected with mycoplasma hyopneumoniae. J Comp Pathol. 2016;154(2–3):165–168. 26922858 10.1016/j.jcpa.2016.01.007

[133] Fourour S, Marois-Créhan C, Martelet L, et al. Intra-species and inter-species differences in cytokine production by porcine antigen-presenting cells stimulated by *Mycoplasma hyopneumoniae*, *M. hyorhinis*, and M. flocculare. Pathogens. 2019;8(1):34. 10.3390/pathogens8010034 PMC6471550 30884861

[134] Woolley LK, Fell S, Gonsalves JR, et al. Evaluation of clinical, histological and immunological changes and qPCR detection of Mycoplasma hyopneumoniae in tissues during the early stages of mycoplasmal pneumonia in pigs after experimental challenge with two field isolates. Vet Microbiol. 2012;161(1–2):186–195. 22863144 10.1016/j.vetmic.2012.07.025

[135] Shen Y, Hu W, Wei Y, et al. Effects of Mycoplasma hyopneumoniae on porcine nasal cavity dendritic cells. Vet Microbiol. 2017;198:1–8. 28061998 10.1016/j.vetmic.2016.11.018

[136] Leal FMDA, Virginio VG, Martello CL, et al. *Mycoplasma hyopneumoniae* and *Mycoplasma flocculare* differential domains from orthologous surface proteins induce distinct cellular immune responses in mice. Vet Microbiol. 2016;50–57. DOI:10.1016/j.vetmic.2016.05.008 27283856

[137] Garcia-Morante B, Segalés J, Fraile L, et al. Potential use of local and systemic humoral immune response parameters to forecast Mycoplasma hyopneumoniae associated lung lesions. PLoS One. 2017;12(4):e0175034. 28380065 10.1371/journal.pone.0175034 PMC5381809

[138] Muneta Y, Minagawa Y, Shimoji Y, et al. Immune response of gnotobiotic piglets against Mycoplasma hyopneumoniae. J Vet Med Sci. 2008;70(10):1065–1070. 18981662 10.1292/jvms.70.1065

[139] Thanawongnuwech R, Thacker EL. Interleukin-10, interleukin-12, and interferon-gamma levels in the respiratory tract following mycoplasma hyopneumoniae and PRRSV infection in pigs. Viral Immunol. 2003;16:357–367. 14583150 10.1089/088282403322396154

[140] Bai F, Ni B, Liu M, et al. Mycoplasma hyopneumoniae-derived lipid-associated membrane proteins induce inflammation and apoptosis in porcine peripheral blood mononuclear cells in vitro. Vet Microbiol. 2015;175(1):58–67. 25481242 10.1016/j.vetmic.2014.11.013

[141] Liu W, Zhou D, Yuan F, et al. Surface proteins mhp390 (P68) contributes to cilium adherence and mediates inflammation and apoptosis in Mycoplasma hyopneumoniae. Microb Pathog. 2019;126:92–100. 30385395 10.1016/j.micpath.2018.10.035

[142] Chandrasekar BS, Yadav S, Victor ES, et al. Interferon-gamma and nitric oxide synthase 2 mediate the aggregation of resident adherent peritoneal exudate cells: implications for the host response to pathogens. PLoS One. 2015;10(6):e0128301. 26029930 10.1371/journal.pone.0128301 PMC4452304

[143] Thom SR, Bhopale VM, Milovanova TN, et al. Nitric-oxide synthase-2 linkage to focal adhesion kinase in neutrophils influences enzyme activity and β2 integrin function. J Biol Chem. 2013;288:4810–4818. 23297409 10.1074/jbc.M112.426353 PMC3576086

[144] Yadav S, Pathak S, Sarikhani M, et al. Nitric oxide synthase 2 enhances the survival of mice during Salmonella Typhimurium infection-induced sepsis by increasing reactive oxygen species, inflammatory cytokines and recruitment of neutrophils to the peritoneal cavity. Free Radic Biol Med. 2018;116:73–87. 29309892 10.1016/j.freeradbiomed.2017.12.032

[145] Imlay JA. Pathways of oxidative damage. Annu Rev Microbiol. 2003;57(1):395–418. 14527285 10.1146/annurev.micro.57.030502.090938

[146] El-Benna J, Hurtado-Nedelec M, Marzaioli V, et al. Priming of the neutrophil respiratory burst: role in host defense and inflammation. Immunol Rev. 2016;273:180–193. 27558335 10.1111/imr.12447

[147] Rose SJ, Bermudez LE, Flynn JL. Mycobacterium avium biofilm attenuates mononuclear phagocyte function by triggering hyperstimulation and apoptosis during early infection. Infect Immun. 2014;82(1):405–412. 24191301 10.1128/IAI.00820-13 PMC3911830

[148] Poderoso JJ, Helfenberger K, Poderoso C. The effect of nitric oxide on mitochondrial respiration. Nitric Oxide. 2019;88:61–72. 30999001 10.1016/j.niox.2019.04.005

[149] Choi SY, Lim JW, Shimizu T, et al. Reactive oxygen species mediate Jak2/Stat3 activation and IL-8 expression in pulmonary epithelial cells stimulated with lipid-associated membrane proteins from Mycoplasma pneumoniae. Inflamm Res. 2012;61(5):493–501. 22270622 10.1007/s00011-012-0437-7

[150] Dusanic D, Bencina D, Oven I, et al. Mycoplasma synoviae induces upregulation of apoptotic genes, secretion of nitric oxide and appearance of an apoptotic phenotype in infected chicken chondrocytes. Vet Res. 2012;43(1):7. 22280251 10.1186/1297-9716-43-7 PMC3293721

[151] Obara H, Harasawa R. Nitric oxide causes anoikis through attenuation of E-cadherin and activation of caspase-3 in human gastric carcinoma AZ-521 cells infected with Mycoplasma hyorhinis. J Vet Med Sci. 2010;72(7):869–874. 20179380 10.1292/jvms.09-0573

[152] Citti C, Nouvel LX, Baranowski E. Phase and antigenic variation in mycoplasmas. Future Microbiol. 2010;5:1073–1085. 20632806 10.2217/fmb.10.71

[153] Moitinho-Silva L, Kondo MY, Oliveira LC, et al. Mycoplasma hyopneumoniae in vitro peptidase activities: identification and cleavage of kallikrein-kinin system-like substrates. Vet Microbiol. 2013;163:264–273. 23421966 10.1016/j.vetmic.2013.01.011

[154] Polosa R, Hasani A, Pavia D, et al. Acute effect of inhaled bradykinin on tracheobronchial clearance in normal humans. Thorax. 1992;47:952–956. 1465754 10.1136/thx.47.11.952 PMC464107

[155] Onaga T. Tachykinin: recent developments and novel roles in health and disease. Biomol Concepts. 2014;5(3):225–243. 25372755 10.1515/bmc-2014-0008

[156] Prod’homme T, Weber MS, Steinman L, et al. A neuropeptide in immune-mediated inflammation, Y? Trends Immunol. 2006;27(4):164–167. 16530483 10.1016/j.it.2006.02.003

[157] Neto JC, Strait EL, Raymond M, et al. Antibody responses of swine following infection with Mycoplasma hyopneumoniae, M. hyorhinis, M. hyosynoviae and M. flocculare. Vet Microbiol. 2014. DOI:10.1016/j.vetmic.2014.08.008 25240775

[158] Yu Y, Wang J, Han R, et al. Evades complement activation by binding to factor H via elongation factor thermo unstable (EF-Tu). Virulence. 2020;11:1059–1074. 32815770 10.1080/21505594.2020.1806664 PMC7549910

[159] Weiler JM, Daha MR, Austen KF, et al. Control of the amplification convertase of complement by the plasma protein beta1H. Proc Natl Acad Sci U S A. 1976;73:3268–3272. 1067618 10.1073/pnas.73.9.3268 PMC431003

[160] Henthorn CR, Chris Minion F, Sahin O. Utilization of macrophage extracellular trap nucleotides by Mycoplasma hyopneumoniae. Microbiology. 2018;164(11):1394–1404. 30383520 10.1099/mic.0.000717

[161] Sirand-Pugnet P, Citti C, Barré A, et al. Evolution of mollicutes: down a bumpy road with twists and turns. Res Microbiol. 2007;158(10):754–766. 18023150 10.1016/j.resmic.2007.09.007

[162] Hames C, Halbedel S, Hoppert M, et al. Glycerol metabolism is important for cytotoxicity of Mycoplasma pneumoniae. J Bacteriol. 2009;191:747–753. 19028882 10.1128/JB.01103-08 PMC2632104

[163] Vilei EM, Frey J. Genetic and biochemical characterization of glycerol uptake in mycoplasma mycoides subsp. mycoides SC: its impact on H(2)O(2) production and virulence. Clin Diagn Lab Immunol. 2001;8:85–92. 11139200 10.1128/CDLI.8.1.85-92.2001 PMC96015

[164] Ferrarini MG, Mucha SG, Parrot D, et al. Hydrogen peroxide production and myo-inositol metabolism as important traits for virulence of Mycoplasma hyopneumoniae. Mol Microbiol. 2018. DOI:10.1111/mmi.13957 29624763

[165] Mucha SG, Ferrarini MG, Moraga C, et al. Mycoplasma hyopneumoniae J elicits an antioxidant response and decreases the expression of ciliary genes in infected swine epithelial cells. Sci Rep. 2020;10:13707. 32792522 10.1038/s41598-020-70040-y PMC7426424

[166] Pan Q, Wang X, Liu T, et al. Mycoplasma hyopneumoniae inhibits porcine beta-defensin 2 production by blocking the unfolded protein response to facilitate epithelial adhesion and infection. Infect Immun. 2020;88(7). DOI:10.1128/IAI.00164-20 PMC7309629 32312764

[167] Gonchoroski T, Virginio VG, Thompson CE, et al. Evolution and function of the Mycoplasma hyopneumoniae peroxiredoxin, a 2-Cys-like enzyme with a single Cys residue. Mol Genet Genomics. 2017;292(2):297–305. 27858147 10.1007/s00438-016-1272-2

[168] Madsen ML, Nettleton D, Thacker EL, et al. Transcriptional profiling of Mycoplasma hyopneumoniae during heat shock using microarrays. Infect Immun. 2006;74(1):160–166. 16368969 10.1128/IAI.74.1.160-166.2006 PMC1346651

[169] Paes JA, Leal Zimmer FMA, Moura H, et al. Differential responses to stress of two Mycoplasma hyopneumoniae strains. J Proteomics. 2019;199:67–76. 30862566 10.1016/j.jprot.2019.03.006

[170] Schafer ER, Oneal MJ, Madsen ML, et al. Global transcriptional analysis of Mycoplasma hyopneumoniae following exposure to hydrogen peroxide. Microbiology. 2007;153(11):3785–3790. 17975087 10.1099/mic.0.2007/011387-0

[171] Ferreira HB, Castro LA. A preliminary survey of *M. hyopneumoniae* virulence factors based on comparative genomic analysis. São Paulo Gene Mol Microbiol. 2007;30(1):245–255.

[172] Bogema DR, Deutscher AT, Woolley LK, et al. Characterization of cleavage events in the multifunctional cilium adhesin Mhp684 (P146) reveals a mechanism by which Mycoplasma hyopneumoniae regulates surface topography. MBio. 2012;3(2): e00282-11. 10.1128/mBio.00282-11 PMC3322551 22493032

[173] Yu Y, Wang H, Wang J, et al. Elongation factor thermo unstable (EF-Tu) moonlights as an adhesin on the surface of mycoplasma hyopneumoniae by binding to fibronectin. Front Microbiol. 2018b;9:974. 29867877 10.3389/fmicb.2018.00974 PMC5962738

[174] Deng X, Zhu Y, Dai P, et al. Three polypeptides screened from phage display random peptide library may be the receptor polypeptide of Mycoplasma genitalium adhesion protein. Microb Pathog. 2018;120:140–146. 29709687 10.1016/j.micpath.2018.04.058

[175] Raymond BB, Tacchi JL, Jarocki VM, et al. P159 from Mycoplasma hyopneumoniae binds porcine cilia and heparin and is cleaved in a manner akin to ectodomain shedding. J Proteome Res. 2013;12:5891–5903. 24195521 10.1021/pr400903s

[176] Minion FC, Jarvill-Taylor K. Membrane-associated hemolysin activities in mycoplasmas. FEMS Microbiol Lett. 1994;116(1):101–106. 8132149 10.1111/j.1574-6968.1994.tb06682.x

[177] Khil PP, Camerini-Otero RD. Over 1000 genes are involved in the DNA damage response of Escherichia coli. Mol Microbiol. 2002;44(1):89–105. 11967071 10.1046/j.1365-2958.2002.02878.x

